# Group problem solving therapy for perinatal depression in primary health care settings in rural Uganda: an intervention cohort study

**DOI:** 10.1186/s12884-021-04043-6

**Published:** 2021-08-25

**Authors:** Juliet E. M. Nakku, Oliva Nalwadda, Emily Garman, Simone Honikman, Charlotte Hanlon, Fred Kigozi, Crick Lund

**Affiliations:** 1 Butabika National Referral Mental Hospital, Kampala, Uganda; 2 Department of psychiatry and mental health, University of Cape Town, Cape Town, South Africa; 3 Alan J Flisher Centre for Public Mental Health, Department of Psychiatry and Mental Health, University of Cape Town, Cape Town, South Africa; 4 Centre for Global Mental Health, Health Service and Population Research Department, Institute of Psychiatry, Psychology and Neuroscience, King’s College, London, UK; 5 Department of Psychiatry, WHO Collaborating Centre for Mental Health Research and Capacity-building, School of Medicine, College of Health Sciences, Addis Ababa University, Addis Ababa, Ethiopia

**Keywords:** Depression, Perinatal, Group problem solving therapy, Primary health care

## Abstract

**Background:**

Perinatal depression is of substantial public health importance in low and middle income countries. The study aimed to evaluate the impact of a mental health intervention delivered by non-specialist health workers on symptom severity and disability in women with perinatal depression in Uganda.

**Methods:**

Pregnant women in the second and third trimester were consecutively screened using the Luganda version of the 9-item Patient Health Questionnaire (PHQ-9). Women who scored ≥5 on the PHQ-9 and who were confirmed to have depression by a midwife were recruited into a treatment cohort and offered a psychological intervention in a stepped care fashion. Women were assessed with PHQ-9 and WHODAS-2.0 at baseline and again at 3 and 6 months after the intervention. Negative regression analysis was done to examine change in PHQ-9 and WHODAS-2.0 scores from baseline to end line. Data were analysed using STATA version 14.

**Results:**

A total of 2652 pregnant women (98.3%) consented to participate in the study and 153 (5.8%) were diagnosed as depressed. Over a quarter (28.8%) reported having experienced physical interpersonal violence (IPV) while (25.5%) reported sexual IPV in the past year. A third (34.7%) of women diagnosed with depression received 4 or more group PST sessions. There was a mean reduction in PHQ-9 score of 5.13 (95%CI − 6.79 to − 3.47, *p* < 0.001) and 7.13 (95%CI − 8.68 to − 5.59, *p* < 0.001) at midline and endline, respectively. WHODAS scores reduced significantly by − 11.78 points (CI 17.64 to − 5.92, *p* < 0.001) at midline and − 22.92 points (CI 17.64 to − 5.92, p < 0.001) at endline. Clinical response was noted among 69.1% (95%CI 60.4–76.6%) and 93.7% (95%CI 87.8–96.8%) of respondents at midline and endline, respectively.

**Conclusion:**

An evidence based psychological intervention implemented in primary antenatal care by trained and supervised midwives in a real-world setting may lead to improved outcomes for women with perinatal depression. Future randomised studies are needed to confirm the efficacy of this intervention and possibility for scale up.

**Supplementary Information:**

The online version contains supplementary material available at 10.1186/s12884-021-04043-6.

## Background

Globally, depression accounts for the largest proportion of the burden associated with mental and neurological disorders in women of child-bearing age [1, 2]. Perinatal depression is the most common mental health complication in the perinatal period [2, 3], with an estimated prevalence of 11.9% [4]. This burden is projected to grow dramatically in the next decade [5]. The prevalence of perinatal depression in Africa is even higher than global estimates. In a systematic review of studies among African perinatal populations, the pooled prevalence of antenatal depression was 26% and for postnatal depression was 17% [6]. In Uganda, the prevalence of perinatal depression ranges between 5 and 10% in pregnant women, [7–9]. In the postpartum period, between 6 and 40% of HIV and non-HIV perinatal populations are at risk for depression [7, 10, 11].

Perinatal depression has adverse effects on the mother, her child and family [12–14]. Previous studies have reported an association of perinatal depression with complications in labour and premature birth, low birth weight, low Apgar scores, diarrhoeal episodes, detrimental changes in fetal heart rate and motor activity as well as increased neonatal intensive care unit admission [12, 15, 16], resulting in compromised child development [3, 17]. Untreated perinatal depression is a risk factor for suicidal ideation and suicide completion in the mother [18, 19], as well as one contributing factor to infanticide [20].

Integration of mental health into primary health care is recommended to address health systems challenges in low-resource settings [21–23]. However, there is still a pervasive lack of routine perinatal mental health care in LMICs, more so in rural settings [13]. The lack of integration between maternal health services, child health services, and mental health services in primary care creates a large gap in the identification and treatment of perinatal mental disorders [24].

Previous studies provided evidence that problem solving therapy (PST) is effective for depression in general primary health care, as well as in perinatal populations [25, 26]. However, studies on how to integrate and evaluate the impact of such evidence-based interventions in real world primary care environments in LMICs are largely missing. The Programme for Improving Mental Health Care (PRIME) was a multi-country study consortium which aimed to generate evidence on the implementation and scaling up of integrated packages of care for priority mental disorders in primary and maternal health care in five LMICs, including Uganda [27]. Under this program, a district Mental Health Care Plan (MHCP) was developed in each country to provide a framework for implementation and evaluation of the impact of integrating evidence-based interventions in a real-world, primary care setting. A detailed description of the development and components of the Uganda district MHCP has been published elsewhere [28].

In this study we aimed to evaluate the impact of a midwife-delivered intervention on depressive symptom severity and disability among pregnant women 6 months after initiation of treatment for perinatal depression in rural Uganda, as part of the PRIME MHCP.

## Methods

The study was conducted in Kamuli district, located in Eastern Uganda. Kamuli’s district has a population of 490,000 rural, poor of little education and engaged is subsistence activity [29]. The district has a high birth rate and population density [30]. More detail on the demographic characteristics of Kamuli has been previously published in the district’s situation analysis [31].

Routine antenatal care in Kamuli is provided at all the primary health care facilities located at different geographical divisions of the district. Kamuli district has 4 sub counties served by 2 (one public and one private) general hospitals (level 5), two level 4 health centers and four level 3 health centers. All these facilities offer both antenatal and postnatal care. The public general hospital is also staffed with a psychiatric clinical officer as well as 2 psychiatric nurses. The district is served by a regional referral hospital in the neighbouring district of Jinja, which is over 60 km away from Kamuli district where complicated cases can be referred to a gynaecologist. A psychiatrist is also available at this referral Hospital. The recruitment of participants in the present study was conducted in five primary care facilities: two level 4 health centres, three level 3 health centres and one general hospital.

We conducted an uncontrolled before-after intervention study with assessments pre-treatment (baseline), 3 months (midline) and 6 months (endline) after baseline or at 2 months postpartum, whichever came first. A single group cohort was selected because we sought to establish the feasibility and acceptability of the intervention, as well as the feasibility of the assessment procedures in this setting, to lay the foundation for a future randomised controlled trial.

Pregnant women were eligible to participate in the study if they were in their second or third trimester of pregnancy, if they resided in the area of study, were aged 18 years or above and spoke the study languages (English or Luganda). Pregnant women were ineligible if in labour, non-residents, and if they had been identified by midwife physical examination as having physical complications related to pregnancy e.g. pre-eclampsia, diabetes or spontaneous abortion.

Sample size was calculated based on a 20% attainment of Clinical response, defined as a 50% reduction of depression symptoms at 6 months, with 90% power, two-sided alpha of 0.05. Sample size calculation was based on a one-sample analysis. An attrition rate of 15–20% at the end of the study was expected. The target sample size was therefore set at 150.

Pregnant women were consecutively screened in the antenatal clinic waiting areas between November 2015 and December 2016. These were recruited from all high-volume public health facilities that offered antenatal and postnatal care. Out of 7 public health facilities in Kamuli district, 5 were considered high volume (with monthly attendance of 500 or more) and were selected. The public facilities (as opposed to private ones) were selected owing to our intention to scale up the intervention in future in public health facilities.

Screening was done by trained non-midwife research assistants using the Luganda version of the 9-item Patient Health Questionnaire (PHQ-9) that had been validated for use in Kamuli in Uganda [32]. Findings from this validation study indicated that the optimum trade-off between sensitivity and positive predictive value (PPV) for probable depression was achieved at a cut-off of ≥5. All women who scored ≥5 on the PHQ-9 in our study were referred to a midwife for confirmation of the depression diagnosis using the World Health Organisation (WHO) Mental Health Gap Action Program Intervention Guide (mhGAP-IG) algorithm [22]. Women who were confirmed to have depression were recruited into a treatment cohort, given information about the aims of the study by the research assistants and invited to provide written consent to participate.

The MHCP was a complex intervention in a real-world setting. The broad components of the MHCP have been previously elaborated [33]. Table 1 below shows the components of the MHCP intervention as it applied to the perinatal setting. This included training of primary care based midwives in assessing, diagnosing and treating perinatal depression in a primary maternal care setting; provision of an adapted evidence based treatment, group based problem solving therapy (PST); providing interpersonal violence support; antidepressant medication for those who had severe symptoms such as suicide as well as supervision and support of the primary care midwives by specialist mental health personnel.

**Table 1:** Components of the mental health care plan for perinatal depression setting in Kamuli district, Uganda

Package area	Awareness and Knowledge Enhancement	Detection	Treatment	Recovery	Program management
Level					
**Health organisation**	● Advocacy ● Perinatal mental health literacy				● Antidepressant drug supply chain management ● Health information system ● Supportive supervision to midwives ● Capacity-building ● Routine monitoring and evaluation
**Primary Health care facility**	In-Service training of midwives in depression care	Depression Screening and assessment	● Antidepressant medication ● Group problem solving therapy		
**Community**	● Community sensitisation through radio ● Engagement of Village Health Team workers.	Community detection of perinatal depression and referral		● Village Health Team Outreach and adherence support ● Intimate Partner violence support	

A stepped care approach was used whereby all women who had PHQ-9 score of 5 or more and were confirmed on mhGAP to be depressed were offered the monthly midwife-led Group Problem Solving Therapy (GPST) sessions during their scheduled antenatal visits. Thirty midwives were trained but twenty participated fully in the study. Some dropped out due to routine transfer to other districts and varying assignments in the facilities. Others oversaw health units with administration and support responsibilities to other midwives and were therefore uninterested citing heavy workloads. Training was done by a psychiatrist, a psychiatric clinical officer and a clinical psychologist. Midwives were also trained in assessment and treatment of depression using the WHO mhGAP algorithms. This training took place from August to September 2015. A 3-month period of embedding the intervention was allowed before recruitment started. Two 2-day refresher training courses were conducted for the midwives at 3-month intervals over the recruitment period. Refresher training was done by the same training team. The trained midwives were similar in the sense that they all held a diploma in midwifery and all worked in a similar public health environment.

Bernal’s framework for adapting psychological interventions in LMIC [34, 35] was used to develop a contextually appropriate version of PST. The Bernal’s framework proposes areas for adaptation including 1) language used e.g., translation; 2) using non-specialist mental health workers as therapists; 3) content addressing day to day stressors; 4) methods e.g., incorporating into existing service schedules, 5) storytelling and use of locally familiar examples; and 6) adapted techniques e.g. minimal or no use of written materials and homework among other adaptations. The group PST was adapted by being offered in local language, task shifting it to primary care midwives, providing in in routine antenatal schedules, including care for common stressors such as interpersonal violence (IPV) support and included psycho-education about maternal depression causes and treatment on top of training in problem solving skills.

Women who were found to score ≥ 5 on the PHQ-9 and reported to have suicidal thoughts or suicidal attempts in the past 2 weeks were referred to a visiting specialist psychiatry clinical officer (PCO) attached to the health facility for medication prescription and monitoring, in addition to group PST. Referrals to the PCO were also initiated for participants who presented with persistent depressive symptoms (PHQ-9 score of ≥5) during the midline and endline assessments. The GPST sessions had an average size of 12–15 mothers. A minimum dose of 4 sessions was offered but women could return for more sessions if they needed to. Sessions took place monthly to coincide with their regular antenatal visits, were conducted in a private convenient space within the health facility premises and lasted for 1.5 to 2 h. In these closed PST groups women were guided through the four steps of PST, including 1) identifying and defining the problem, 2) weighing alternative solutions, 3) choosing and implementing the most feasible solution, and 4) evaluating the outcome. In each session, one chosen problem per woman was discussed. Often these problems were common to a number of women in the group and would therefore be handled collaboratively and reported back verbally at the next session. No written homework was expected. IPV support was provided by a team from the Uganda Women’s Network (UWONET), a local non-government organisation providing such support who attended at least one group session to provide information and support to the women.

Monthly supportive supervision was conducted by a visiting mental health specialist (psychiatric clinical officer (PCO) or psychologist) to support midwives in addressing capacity needs and gaps. The supervisors (who were part of the training team) met with the midwives at the clinics to ensure that accurate/correct confirmation of diagnosis was occurring. From time to time, they sat in on the group sessions as observers to identify areas for support to the midwives regarding PST process which formed the content of the refresher trainings. They also provided support in dealing with any challenges the midwives were encountering along the way.

Baseline questionnaires were administered at the participant’s homes within 7 days of recruitment. Midline (3 months from baseline) and endline (6 months from baseline) assessments were also done at home with a window of 2 weeks before or after the appointed date for the interview. All data collection was conducted on an Android mobile device linked to the Mobenzi online application.

Demographic and socio-economic characteristics were assessed using questions adapted from the Uganda Demographic and Health Surveys [32]. Variables assessed included age, sex, education attainment, occupation as well as economic status, employment status and income. History of physical and sexual abuse was assessed using the Abuse Assessment Screen and alcohol use was assessed using the Alcohol Use Disorder Identification Test (AUDIT). Further variables assessed included perceived social support (Oslo Social Support Scale) and accommodation. The full questionnaire can be found in supplementary file 2.

The Primary outcome variables were clinical response and disability score. Clinical response was defined as at least a 50% improvement in the depression symptom score on the PHQ-9. The PHQ-9 has previously been validated in African populations including pregnant women in Ethiopia and Uganda [32, 36]. The nine items of the PHQ-9 are based on the nine diagnostic criteria for major depressive disorder in the DSM-IV [37]. Each item is scored on a Likert scale with symptoms rated as 0 (not at all), 1 (several days), 2 (more than half the days) and 3 (nearly every day) during the past 2 weeks. The sum of the scores indicates whether the respondent has mild (PHQ score 5–9), moderate (PHQ score 10–14) or severe (PHQ score > 15) levels of depressive symptoms.

The World Health Organization (WHO) disability assessment schedule 2.0 (WHODAS 2.0) [38] was used to assess disability. The WHODAS 2.0 is a generic assessment tool for health and disability, which is appropriate for use across cultures in adult populations and was found to be reliable across 19 countries. Each item is measured on a 5-point Likert scale and item scores are summed up to give the degree of functional limitation. Greater WHODAS scores indicate greater functional impairment [39].

To screen for alcohol use disorders (AUD), the 10-item AUDIT was used. The AUDIT was designed to identify hazardous drinkers, harmful drinkers and people with alcohol dependence. The tool was designed by WHO and can be used in both community and clinical settings. Each item concerns the frequency of different drinking behaviours and consequences over the past 12 months. Responses are scored from 0 (never occurs) to 4 (daily). The sum of scores indicate whether the respondent engages in hazardous (score 8–15), harmful (score 16–19) or dependent (score ≥ 20) drinking behaviours, using cut-offs defined by the WHO [40, 41].

In Uganda, the AUDIT has not been validated in perinatal populations but has been shown to have good psychometric properties in post conflict populations in the northern part of the country [42]. The later study elucidated the performance of the AUDIT at different cut off points (≥3, ≥5, ≥8) for problematic drinking and recommended a cut off of 3 or more for screening in population studies. Our study opted to use a cut off of 8 in accordance with the cut-point used other LMICs [41, 42], in order to pick up problematic drinking with more certainty.

The Oslo Social Support Scale (OSSS-3) consists of three items and was used to assess participants’ perceived level of social support. It has been recommended for epidemiological and population-based surveys. The sum score ranges from 3 to 14, with high values representing strong levels and low values representing poor levels of social support. The OSSS-3 sum score can be operationalized into three broad categories of social support [43] namely poor social support (score 3–8), moderate social support (score 9–11) and strong social support (score 12–14). Although the OSSS-3 tool has not been validated in Ugandan, it has been validated among Nigerian students and was found to have good discriminate validity [44].

The Abuse Assessment Screen was developed as a clinical tool to assess frequency, severity and perpetrators of violence against women [45]. It is short and easy to administer. It assesses mainly physical and sexual violence. Although it has not been validated in African populations, it has been found useful and easy to use in other populations around the world [46].

Data were migrated from the Mobenzi online application to STATA version 14 where they were analysed. Data were summarized using frequency tables. The two-sided ᾳ-level was set at 0.05. Reduction of at least 50% in PHQ-9 scores from baseline to endline (clinical response), our primary outcome, is reported. PHQ-9 scores were not normally distributed, so differences in scores across demographic categories were assessed using non-parametric tests, namely the Mann-Whitney U Test or the Kruskal Wallis test. Univariate negative binomial regression analysis was conducted to assess change in PHQ-9 and WHODAS from baseline to midline and from baseline to endline. Change in PHQ-9 scores from baseline to endline across demographic characteristics (education, employment status and food insecurity), perceived social support and IPV [47] was also assessed. This was done using univariate binomial regression models, this time including each demographic variable as an interaction term. For this, education was binarized into uneducated vs. educated, and social support binarized into low/moderate support (OSSS-3 score < 12) vs high support (OSSS-3 score > 11). Change in PHQ-9 scores by number of sessions attended was also assessed using the same analysis.

## Results

### Respondent characteristics at baseline

**Fig. 1:**
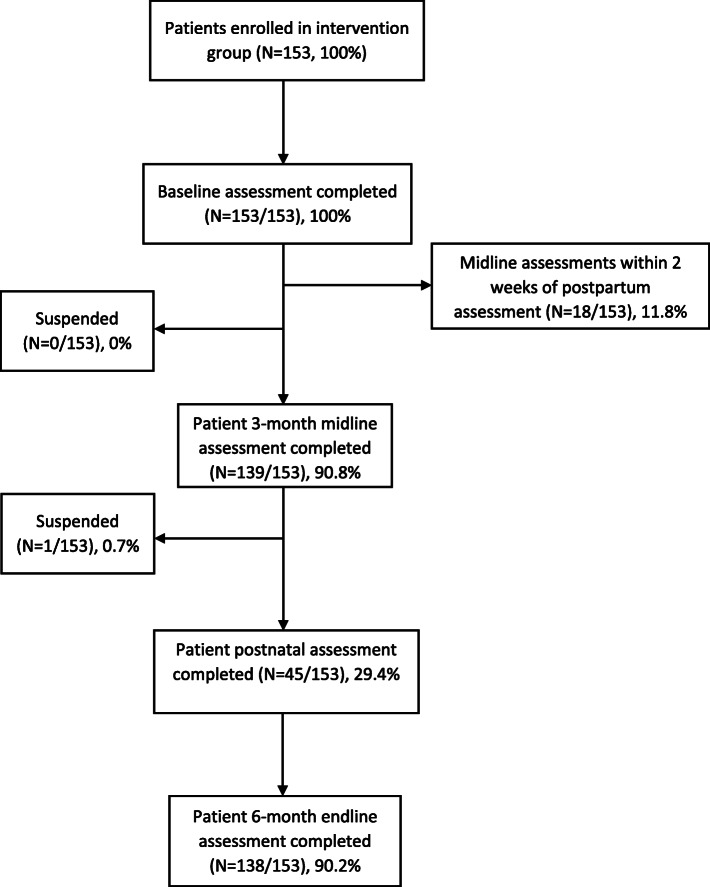
Perinatal Depression Cohort Recruitment Flow Diagram

A total of 2698 pregnant women were approached and 2652 (98.3%) consented to participate in the study. Of these, 153 (5.8%) screened positive and were diagnosed as depressed by the midwife and so were included in our study. Of these, 139 (90.8%) were followed-up at 3 months and 138 (90.2%) were followed-up at 6 months. Fig. 1 demonstrates the flow of participants in a consort diagram. The participants lost to follow-up did not present any demographic or clinical differences from those who were assessed at 6 months.

Baseline characteristics of the study sample are shown in Table 2 below. The largest percentage of women (33.3%) were aged between 26 and 30 years. Only 19% were above the age of 30 years and about half (56.2%) were in the 2nd trimester of pregnancy at recruitment. The majority of women (79%) reported being married or being in a stable relationship with a male partner. Only 18% of the respondents had completed secondary school education, and a third (*n* = 53, 34.6%) reported being illiterate. About two-thirds of women in the study did not have any form of employment, with 17% reported having food insecurity. Over a quarter of participants had experienced physical IPV (*n* = 44, 28.8%) or sexual IPV (*n* = 39, 25.5%) in the past year.

**Table 2:** Demographic characteristics of the cohort sample

	N	%	Median (IQR) PHQ-9 score	*χ or U*	*p*
Age (years)					
18–20	37	24.2	8 (6–10)	0.983	0.805
21–25	36	23.5	8.5 (7–10)		
26–30	51	33.3	8 (7–10)		
> 30	29	19.0	8 (7–10)		
Trimester at recruitment					
Second	86	56.2	8 (7–10)	−0.808	0.419
Third	67	43.8	8 (7–10)		
Marital status					
No partner	32	20.9	8 (7–12)	1.241	0.215
Has a partner	121	79.1	8 (7–10)		
Educational level					
Uneducated/illiterate	53	34.6	9 (7–10)	0.357	0.836
Primary school	72	47.1	8 (7–10)		
Secondary school	28	18.3	8.5 (7–10)		
Employment					
Not employed	104	68.0	9 (7–10)	2.260	0.024
Employed	49	32.0	8 (7–9)		
Household food insecurity					
No	127	83.0	8 (7–10)	−2.111	0.035
Yes	26	17.0	9 (7–13)		
Interpersonal violence in past year					
Physical	44	28.8	9 (7.5–11.5)	−2.260	0.024
Sexual	39	25.5	8 (7–10)	−0.221	0.826
Social support (OSSS-3)					
Poor	20	13.1	9 (7–10)	0.203	0.903
Moderate	59	38.6	9 (7–10)		
Strong	74	48.4	8 (7–10)		

*IQR* Inter-quartile range; *PHQ-9* Patient Health Questionnaire – 9 item; *SD* standard deviation; *WHODAS* WHO Disability Assessment Schedule.

The median PHQ-9 score at baseline was 8 (interquartile range (IQR) = 7–10). Participants who were unemployed, who were food insecure and who reported physical IPV all had significantly greater PHQ-9 scores at baseline compared to those who were employed, food secure or did not experience physical IPV. However, there was no significant association between baseline PHQ-9 scores and any of the other demographic characteristics, or with sexual IPV. The median (IQR) scores at baseline for the WHODAS and OSS-3 were 31 [19–32, 34–43] and 11 [10–13], respectively. A total of 20 participants (13.1%) reported having poor perceived social support. Nearly all participants reported not drinking alcohol at baseline (*n* = 136, 93.8%), so AUDIT score was not included in the subsequent analyses.

The total number of sessions attended by the respondents ranged from 0 to 7. A third of participants received 4 or more sessions (*n* = 51, 34.7%). A minority of participants only received 1 session (*n* = 12, 8.2%). Twenty-seven (27, 18.4%) participants received no sessions at all. Only 5 (3.3%) of the participants received antidepressants.

#### Impact of the intervention on individual depression and functioning outcomes

The mean PHQ-9 score at baseline was 9.0 (SD 2.62). There was a significant reduction in mean PHQ-9 score at midline (β = − 5.13, CI − 6.79 to − 3.47, *p* < 0.001) as well as at endline (− 7.13, CI − 8.68 to − 5.59, *p* < 0.001) (See Table 3). Clinical response, defined as 50% or more reduction in symptom score PHQ-9 was observed among 69.1% (95%CI 60.38–76.56%) and 93.7% (95%CI 87.84–96.84%) of respondents at midline and endline, respectively. Similarly, the mean WHODAS score changed significantly from 30.5 (13.91) at baseline by − 11.78 points (CI 17.64 to − 5.92, *p* < 0.001) at midline and − 22.92 points (CI 17.64 to − 5.92, *p* < 0.001) at endline.

**Table 3:** Impact of a midwife delivered Group PST on individual level outcomes (Negative binomial regression)

	N	Mean (SD)	Mean change from BL (*β)*	95%CI	*P*
Baseline					
PHQ-9 score	153	9.0 (2.62)	–	–	–
WHODAS	153	30.5 (13.91)	–	–	–
Midline (3 months)					
PHQ-9 score	139	3.8 (2.86)	−5.13	−6.79 to −3.47	< 0.001
WHODAS	139	18.7 (13.12)	−11.78	−17.64 to −5.92	< 0.001
Endline (6 months)					
PHQ-9 score	138	1.8 (2.03)	−7.13	−8.68 to −5.59	< 0.001
WHODAS	138	7.6 (8.48)	−22.93	−17.64 to −5.92	< 0.001

*BL* Baseline *PHQ-9* Patient Health Questionnaire – 9 item; *SD* standard deviation; *WHODAS* WHO Disability Assessment Schedule.

### Factors associated with change in PHQ-9 scores at endline

Demographic and intervention characteristics associated with the outcome on PHQ-9 scores from baseline to endline are shown in Table 4. Neither education, employment status nor food insecurity were associated with change in PHQ-9 scores at endline. The change in PHQ-9 scores from baseline to endline also did not differ between participants who reported physical IPV (β = − 1.26, 95%CI − 4.80 to 2.28) or sexual IPV (β = − 6.91, 95%CI − 3.28 to 3.88) compared to those who did not report any IPV at baseline. Also, participants with poor or moderate social support at baseline did not report a different change in PHQ-9 scores compared to those with strong (β = − 0.17, 95%CI − 3.26 to 2.92) social support. Finally, change in PHQ-9 scores from baseline to endline was not different between participants who did not attend any sessions compared to those who attended 1–3 sessions (β = − 0.59, 95%CI − 4.85 to 3.68) or 4–7 sessions (β = 0.80, 95%CI − 5.27 to 3.67).

**Table 4:** Unadjusted regression models assessing factors associated with change in PHQ-9 scores at endline (6 months)

	N	Mean change (95%CI) from BL	*Difference in mean change*	95%CI	*p*
Demographic **characteristics**					
Educational level					
Uneducated/illiterate	48	−7.12 (−9.70 to − 4.54)	ref	–	–
Educated (primary or secondary school)	66	−7.14 (−9.39 to − 4.89)	− 0.02	− 3.44 to 3.41	0.992
Employment					
Not employed	96	−7.83 (−9.75 to − 5.91)	ref	–	–
Employed	42	−5.61 (−8.22 to − 2.99)	2.22	−1.02 to 5.46	0.179
Household food insecurity					
No	116	−6.86 (−8.52 to −5.21)	ref	–	–
Yes	22	−8.47 (−12.66 to −4.29)	1.61	−6.11 to 2.89	0.484
Physical IPV in past year					
No	94	−6.73 (−8.52 to −4.94)	ref	–	–
Yes	34	−8.03 (− 11.08 to −4.97)	−1.26	−4.80 to 2.28	0.487
Sexual IPV in past year					
No	95	−7.17 (−8.95 to −5.39)	ref	–	–
Yes	35	−6.91 (−10.02 to − 3.80)	0.30	−3.28 to 3.88	0.869
Social support (OSS-3)					
Poor/moderate	70	−7.05 (−9.20 to −4.90)	Ref	–	–
Strong	68	−7.22 (−9.44 to −5.00)	− 0.17	−3.26 to 2.92	0.914

## Discussion

In this study we aimed to evaluate the impact of a midwife delivered intervention on perinatal depression symptom severity and functional disability among pregnant women 6 months after initiation of treatment in Kamuli District in rural Uganda. Our results showed significant improvement in the clinical and functional outcomes of perinatally depressed women after 6 months. Clinical response was noted in 93.7% of individuals attending PST group sessions. Depression care has been previously integrated in PHC settings and found to be effective in reducing morbidity [25, 48, 49]. However, it has not been widely studied in perinatal settings in LMICs. This study is the first of its kind in a real world primary care perinatal setting in Uganda.

Group PST has been shown to be effective in treatment of depression and other common mental disorders in primary care LMIC [50]. For example, Chibanda and others (2014) found that it was feasible and acceptable to provide PST as a low-cost intervention delivered by lay health workers for perinatal women in primary health care [51]. Given the relatively good uptake of the group intervention in our study, the findings imply that GPST may not only be feasible to integrate in primary perinatal services but may also be acceptable to perinatal women in Uganda.

In this study, we provided a pragmatic psychological intervention, adapted group PST, delivered monthly at the time when mothers were scheduled to return to the clinic for antenatal checks. The relevance of the intervention was enhanced by adding in IPV support sessions. The latter could have enabled women to find a safe space to talk about violence in their life and to rest in the knowledge of where and how to get support. There is a lack of evidence regarding the effect of IPV enhanced PST in LMICs, especially in Africa. Although a previous metanalysis of 15 studies in LMICs did not show any relationship between psychological intervention outcomes and presence or absence of IPV in women with common mental disorders including depression [47], other studies in Iran and Pakistan showed possible beneficial effects with IPV enhanced narrative exposure and group cognitive behavioral therapy respectively. Although we did not find a significant association between the women’s experience of IPV and their clinical outcomes we are unable to conclude on whether our adapted PST enhanced with IPV support impacted clinical response. Whether the presence or absence of IPV impacts response to psychological therapy for women with perinatal depression in low resource settings like Uganda needs to be further evaluated.

Social determinants for depression such as level of education, employment status, food insecurity, physical IPV, sexual IPV and status or level of social support have been deemed important to address when providing perinatal depression care in low resource settings [52]. In our study these factors were not significantly associated with change in PHQ-9 scores among our perinatal women. It is possible that the factors associated with the change in PHQ-9 scores in our sample were multiple and complex and not easy to isolate in this study. Additionally, previous researchers have suggested that adaptation of PST to suit local settings may improve both intervention acceptability and outcomes [25, 53]. The adaptation of our PST intervention in this study was possibly an enabler, making our treatment more acceptable and ensuring improved outcomes for depressed perinatal women following Group PST.

The adaptations in our study included providing the PST in groups rather than individually. The group mode of PST delivery may have provided mothers with additional peer support and opportunities to practice interpersonal skills which may explain the improved PHQ-9 and WHODAS scores at endline. Conducting sessions in the local language, well understood by the majority of the population, enabled the women to engage with therapy while scheduling sessions at the time of the scheduled antenatal visits (usually monthly) rather than weekly, may have improved accessibility. One would argue that such a low intensity intervention would not be effective to produce the intended outcomes in symptom severity and function. In fact in a trial of a psychological intervention involving PST and other methods for perinatal women in Khayelitsha, South Africa was found to have no effect on depression outcomes [54]. This prompted the researchers to call for reflection on the strategy of low intensity task shifted psychological interventions in low resource settings. Our pragmatic low intensity intervention, indicates that GPST can be feasibly and acceptably delivered by trained and supervised primary care midwives to depressed perinatal women, but further research using a randomized controlled trial design is needed to test its effectiveness.

Intimate partner violence has previously been documented as a causal factor for depression among pregnant women [55, 56], a key factor for persistence of depression in the perinatal period [55, 56] and a barrier for attendance and active participation in group therapeutic interventions [57–59]. Intimate partner violence was a common problem reported among the perinatal women in this study. Enhancing Group PST with IPV support therefore may have provided the needed resources to help women deal with this problem and hence improve treatment outcomes. A previous review of studies on effect of psychological interventions among women with common mental disorders showed that having suffered interpersonal violence did not influence the women’s response to treatment [47]. In this study, we were unable to conclude whether the IPV intervention had an effect on perinatal depression or its response to treatment, given the limited IPV intervention. Nevertheless this may be an important component to include in a future trial, as part of an integrated package of care.

More research is needed to determine whether and how IPV impacts response to psychological interventions in women with perinatal depression. This study, nonetheless, offers optimism about a feasible and acceptable treatment option for depressed perinatal women in LMICs.

There are barriers to integrating mental health into primary care that have been highlighted by previous authors [48] which may apply to perinatal settings as well. Our multi component MHCP attempted to address some of these barriers such as lack or inadequate staffing with mental health specialists, low or no mental health literacy among primary health care workers, lack of access to psychological treatments in primary care and stigma associated with a diagnosis such as depression. Working with midwives and enhancing their ability to facilitate Group PST sessions may have not only increased the accessibility and adherence to the Group PST sessions but also may have reduced self and social stigma often associated with mental health care.

Primary health care workers including midwives and nurses are the first and often the only point of health care contact for the majority of women seeking perinatal services in Uganda. By ensuring training of the midwives, we improved their perinatal depression literacy and therefore enhanced depression identification and chance of treatment. However, this would not have been sufficient to produce outcomes in the perinatal women. Previous studies suggest the importance of supervision and support to non-specialist therapists following training in evidence-based practice for mental health care [52, 60, 61]. A lack of this may lead to deterioration of knowledge and therapeutic skills as well as low therapist confidence and fidelity to a therapy. This may result in less than expected clinical outcomes for patients. Supportive supervision and the practical refresher trainings provided to midwives in Kamuli may have been a key motivation to midwives to continue providing GPST and improved quality of the intervention which led to positive clinical outcomes. Further work is needed to clarify what content, quality, method and dose of supportive supervision is required for trained non specialist health workers and how this influences sustainability of care and patient outcomes.

Limitations in this study included the fact that the population studied, though typical of many rural populations in Uganda, may not be representative of all Ugandan perinatal women. Further still this was not a controlled study, but rather a pragmatic intervention study in a real world primary care setting. It is possible that the positive outcomes could have been due to spontaneous remission, regression to the mean or related to another unmeasured intervention or environmental trends. Further randomized controlled studies are needed to confirm the efficacy and effectiveness of this low intensity integrated task shifted GPST in a low resource primary perinatal care setting in Uganda. Nonetheless, this pilot provides useful information about the feasibility and acceptability of GPST and the study instruments that were used in this context. It also provides a tested platform for a future randomized controlled trial.

## Conclusion

These limitations above notwithstanding, this study provides much needed preliminary evidence that a midwife delivered group problem solving therapy is feasible and acceptable and may lead to significant reduction in depression symptom severity and functional disability at 6 months among women with perinatal depression in primary care in low income settings. This has implications for improvement of access to maternal depression care in perinatal care settings in LMICs where there is, usually, a severe lack of mental health specialists. The effect of IPV on perinatal depression severity and response to the GPST could not be conclusively elucidated in this study. Further research is needed to clarify the effect of IPV on perinatal depression and on treatment response. We also recommend that more rigorous randomized controlled studies be carried out to confirm the efficacy and effectiveness of GPST in treatment of perinatal depression in such low resourced primary care settings.

## Supplementary Information

**Additional file 1:.** Prime Cohort Questionnaire. The file comprises of the study tool used during the PRIME cohort surveys at baseline, Midline (at 3 months) and Endline (at 6 months).

## Data Availability

The datasets used and/or analysed during the current study are available from the corresponding author on reasonable request.

## Abbreviations

PST: Problem solving therapy
IPV: Intimate partner violence
LMICs: Low and middle income countries
MHCP: Mental health care plan
PHQ: Patient health questionnaire
WHODAS: World health organisation disability assessment scale
AUDIT: Alcohol use disorder identification test
OSSS: Oslo social support scale
DSM: Diagnostic and statistical manual
UWONET: Uganda women’s network
PRIME: Programme for improved mental health care
