# Supplementary material for: Group problem solving therapy for perinatal depression in primary health care settings in rural Uganda: an intervention cohort study
Source: BMC Pregnancy Childbirth. 2021 Aug 25;21:584. doi: 10.1186/s12884-021-04043-6 (PMC8386083; doi:10.1186/s12884-021-04043-6)
Supplement: Supplementary file 1 — Additional file 1:. Prime Cohort Questionnaire. The file comprises of the study tool used during the PRIME cohort surveys at baseline, Midline (at 3 months) and Endline (at 6 months). [file 12884_2021_4043_MOESM1_ESM.pdf]

PRIME Cohorts (English)

Last Modified by: Emily Baron **on** 17 Nov 2016 13:51:51 **Revision number:** 1728 **Field Count:** 499

Section 1. Variables

1.1 Required field

PRESS NEXT TO BEGIN

1.2 Total of AUD2 and AUD3

Numeric

This field is not displayed on the handset

1.3 Number Health Providers

Numeric

This field is not displayed on the handset

1.4 Participant-proxy

Numeric

This field is not displayed on the handset, Value: 4

1.5 twoweek

Numeric

This field is not displayed on the handset

1.6 onemonth

Numeric

This field is not displayed on the handset

1.7 threemonth

Numeric

This field is not displayed on the handset

1.8 sixmonth

Numeric

This field is not displayed on the handset

1.9 oneyear

Numeric

This field is not displayed on the handset

Prerequisites
Skip when ***Participant-proxy (1.4)*** **Equals '4'** OR
Skip when ***Participant-proxy (1.4)*** **Not Equal '4'**

1.10 firstsecond

Firstsecond

Expects a single option response (optional)

- first [1]
- second [2]
- third [3]
- fourth [4]
- fifth [5]
- sixth [6]
- seventh [7]
- eighth [8]
- ninth [9]
- tenth [10]

Prerequisites
Skip when ***Participant-proxy (1.4)*** **Equals '4'** OR
Skip when ***Participant-proxy (1.4)*** **Not Equal '4'**

1.11 currency

currency units

Expects a single option response (**required**)

- Birr [1]
- Indian Rupees [2]
- Nepali Rupees [3]
- Rand [4]
- Shillings [5]

1.12 setoneyear

Operator

This field is not displayed on the handset, Operator: Set(**oneyear (1.9)** , DATEADD(DATENO) ,-365))

1.13 setthreemonth

Operator

This field is not displayed on the handset, Operator: Set(**threemonth (1.7)** , DATEADD(DATENO) ,-90))

1.14 setsixmonth

Operator

This field is not displayed on the handset, Operator: Set(**sixmonth (1.8)** , DATEADD(DATENO) ,-180))

1.15 setonemonth

Operator

This field is not displayed on the handset, Operator: Set(**onemonth (1.6)** , DATEADD(DATENO) ,-30))

1.16 settwoweeks

Operator

This field is not displayed on the handset, Operator: Set(**twoweek (1.5)** , DATEADD(DATENO) ,-14))

1.17 numberoffeatures

Numeric

This field is not displayed on the handset

1.18 TotalPHQ

Numeric

This field is not displayed on the handset

Section 2. pid

2.1 pid

[Enter participant ID number]

Expects a numeric response (**required**)

2.2 pid2

[Re-enter participant ID number]

Expects a numeric response (**required**)

Prerequisites
Skip when ***pid (2.1)*** **Equals 'q389480'**

2.3 pid_error

[PID does not match. Try again.]

Expects a single option response (**required**)

- Try again [0]

Branches

If response **Equals 'Try again [0]'** then skip to ***pid (2.1)***

Section 3. Introduction

3.1 country

PRIME country

Expects a single option response (**required**)

- Ethiopia - Sodo [1]
- India - Sehore [2]
- Nepal - Chitwan [3]
- South Africa - Dr KK [4]
- Uganda - Kamuli [5]

3.2 cohort

What cohort?

Expects a single option response (**required**)

- depression [1]
- maternal depression [2]
- alcohol [3]
- psychosis [4]
- epilepsy [5]

Prerequisites
Skip when ***cohort (3.2)*** **Equals 'maternal depression [2]'**

3.3 participant

Who is the respondent for this interview?

Expects a single option response (**required**), Default: Caregiver

- Caregiver [1]
- Patient [2]

Prerequisites
Skip when ***cohort (3.2)*** **Equals 'depression [1]'** OR
Skip when ***cohort (3.2)*** **Equals 'maternal depression [2]'** OR
Skip when ***cohort (3.2)*** **Equals 'alcohol [3]'**

3.4 proxy

Who will complete the 'Health care use and costs' and 'Medication' sections?

Expects a single option response (**required**), Default: Caregiver

- Caregiver [1]
- Participant [2]

3.5 ReprocessParticipant-Proxy

Operator

This field is not displayed on the handset, Operator: Reprocess(**Participant-proxy (1.4)**)

Prerequisites
Skip when ***cohort (3.2)*** **Equals 'depression [1]'** OR
Skip when ***cohort (3.2)*** **Equals 'maternal depression [2]'** OR
Skip when ***cohort (3.2)*** **Equals 'alcohol [3]'**

3.6 SetParticipant-Proxy

Operator

This field is not displayed on the handset, Operator: Set(**Participant-proxy (1.4)** , Sum of (Q 37634) , Q **proxy (3.4)**))

3.7 visit

What cohort study visit?

Expects a single option response (**required**)

- Baseline [1]
- Midline [2]
- Endline [3]
- 2-month postpartum [Uganda only] [4]

Prerequisites
Skip when ***cohort (3.2)*** **Not Equal 'maternal depression [2]'** OR
Skip when ***visit (3.7)*** **Not Equal 'Baseline [1]'**

3.8 negmd

Did the woman screen negative or positive on the PHQ-9?

Expects a single option response (**required**)

- Negative [not recruited] [0]
- Positive [1]

Prerequisites
Skip when ***cohort (3.2)*** **Not Equal 'maternal depression [2]'** OR
Skip when ***visit (3.7)*** **Not Equal 'Baseline [1]'**

3.9 diagconfirmed

Was a diagnosis of maternal depression confirmed by the midwife?

Expects a single option response (**required**)

- No [0]
- Yes [1]

Prerequisites
Skip when ***country (3.1)*** **Equals 'Nepal - Chitwan [3]'** OR
Skip when ***country (3.1)*** **Equals 'South Africa - Dr KK [4]'** OR
Skip when ***country (3.1)*** **Equals 'Uganda - Kamuli [5]'**

3.10 section

[TEMPORARY VARIABLE: What section do you want to pilot test?]

Expects a single option response (**required**)

- Demographics [1]
- Clinical history [2]
- Short inventory of problems [3]
- PHQ9 [4]
- Suicidality [5]
- Epilepsy [6]
- Maternal health [7]
- WHODAS [8]
- Inpatient care [9]
- Outpatient care [10]
- PACIC [11]
- Medications [12]
- Community-group interventions [13]
- Economic activity [14]
- OSLO [15]
- Disclosure-patient [16]
- DISC12 [17]
- Severe adverse events [18]
- WHOFIS-Stigma [19]
- Caregiver work burden [20]
- WHOFIS-Impact [21]
- Human rights abuses [22]

Branches

If response **Equals 'Demographics [1]'** then skip to ***sex (4.1)***
If response **Equals 'Clinical history [2]'** then skip to ***starty (5.1)***
If response **Equals 'Short inventory of problems [3]'** then skip to ***sip Instructions (8.1)***
If response **Equals 'PHQ9 [4]'** then skip to ***PHQ-9 Instruction (9.1)***
If response **Equals 'Suicidality [5]'** then skip to ***SUITHINK (10.2)***
If response **Equals 'Epilepsy [6]'** then skip to ***epilastd (11.3)***
If response **Equals 'Maternal health [7]'** then skip to ***pregstatus (12.1)***
If response **Equals 'WHODAS [8]'** then skip to ***WHO Disability Assessment Instruction 1 (13.1)***
If response **Equals 'Outpatient care [10]'** then skip to ***outpatient instruction (16.2)***
If response **Equals 'PACIC [11]'** then skip to ***pacic instructions 1 (18.1)***
If response **Equals 'Medications [12]'** then skip to ***rx_instructions (19.2)***
If response **Equals 'Community-group interventions [13]'** then skip to ***groupinterventions (22.1)***
If response **Equals 'Economic activity [14]'** then skip to ***empever (37.8)***
If response **Equals 'OSLO [15]'** then skip to ***oslo Instruction 1 (38.1)***
If response **Equals 'Disclosure-patient [16]'** then skip to ***pdisclosure (40.1)***
If response **Equals 'DISC12 [17]'** then skip to ***DISC Instruction (41.1)***
If response **Equals 'Inpatient care [9]'** then skip to ***ipd12mo (14.3)***
If response **Equals 'WHOFIS-Stigma [19]'** then skip to ***Stigma Instructions 1 (42.1)***
If response **Equals 'Caregiver work burden [20]'** then skip to ***reducework (43.1)***
If response **Equals 'Human rights abuses [22]'** then skip to ***hrcgever (45.1)***
If response **Equals 'WHOFIS-Impact [21]'** then skip to ***fisinstruction (44.1)***

Section Prerequisites
Skip when ***visit (3.7)*** **Not Equal'Baseline [1]'**

Section 4. Demographics

4.1 sex

[Interviewee sex]

Expects a single option response (**required**)

- Male [0]
- Female [1]

4.2 age

How old are you? (completed years)

Expects a numeric response (**required**)

Constraints

Response must be ***Greater Than or Equal'16'***

Prerequisites
Skip when ***country (3.1)*** **Equals 'India - Sehore [2]'**

4.3 edu

What is the highest level of education you have completed?

Expects a single option response (**required**)

- Uneducated [1]
- Non-formal [2]
- Primary school [3]
- Secondary school [4]
- College/University [5]

Prerequisites
Skip when ***country (3.1)*** **Not Equal 'India - Sehore [2]'**

4.4 edu_in

How many years of education have you completed?

Expects a numeric response (**required**)

4.5 marit

What is your marital status?

Expects a single option response (**required**)

- Single [1]
- Married [2]
- Divorced [3]
- Widowed [4]
- Married but not living together [5]

Prerequisites
Skip when ***sex (4.1)*** **Equals 'Male [0]'**

4.6 preg

Are you pregnant?

Expects a single option response (**required**)

- No [0]
- Yes [1]
- Not applicable [66]
- Don't know [888]

Prerequisites
Skip when ***preg (4.6)*** **Not Equal 'Yes [1]'** OR
Skip when ***marit (4.5)*** **Not Equal 'Married [2]'**

4.7 pregmon

How many months pregnant?

Expects a numeric response (**required**)

Prerequisites
Skip when ***country (3.1)*** **Not Equal 'South Africa - Dr KK [4]'** AND
Skip when ***marit (4.5)*** **Equals 'Single [1]'**

4.8 kids

Do you have children?

Expects a single option response (**required**)

- No [0]
- Yes [1]

Prerequisites
Skip when ***kids (4.8)*** **Not Equal 'Yes [1]'**

4.9 youngyears

How old is your YOUNGEST child? [Enter age in YEARS] [If age is <2 years, then enter 0]

Expects a numeric response (**required**)

Constraints

*Response must be **Not Equal'1'***

Prerequisites
Skip when ***youngyears (4.9)*** **Greater Than '1'** OR
Skip when ***kids (4.8)*** **Not Equal 'Yes [1]'**

4.10 youngmonths

How old is your youngest child? [Enter age from 0 to 23 months]

Expects a numeric response (**required**)

Constraints

*Response must be **between'1' and '23'** OR*

*Response must be **Equals'88'***

Prerequisites
Skip when ***country (3.1)*** **Not Equal 'India - Sehore [2]'**

4.11 rel_in

What is your religion?

Expects a single option response (**required**)

- Moslem [2]
- Hindu [5]
- Buddhist/Neo-Buddhist [6]
- Christian [7]
- Sikh [8]
- Jain [9]
- Other [Specify] [77]

Prerequisites
Skip when ***country (3.1)*** **Not Equal 'South Africa - Dr KK [4]'**

4.12 rel_sa

What is your religion?

Expects a single option response (**required**)

- Moslem [2]
- Hindu [5]
- Buddhist [6]
- Christian [7]
- Other [77]

Prerequisites
Skip when ***country (3.1)*** **Not Equal 'Uganda - Kamuli [5]'**

4.13 rel

What is your religion?

Expects a single option response (**required**)

- Catholic [1]
- Moslem [2]
- Protestant [3]
- Hindu [5]
- Other [Specify] [77]

Prerequisites
Skip when ***rel_in (4.11)*** **Equals 'Moslem [2]'** OR
Skip when ***rel_in (4.11)*** **Equals 'Hindu [5]'** OR
Skip when ***rel_in (4.11)*** **Equals 'Buddhist / Neo-Buddhist [6]'** OR
Skip when ***rel_in (4.11)*** **Equals 'Christian [7]'** OR
Skip when ***rel_in (4.11)*** **Equals 'Sikh [8]'** OR
Skip when ***rel_in (4.11)*** **Equals 'Jain [9]'** OR
Skip when ***rel_sa (4.12)*** **Equals 'Moslem [2]'** OR
Skip when ***rel_sa (4.12)*** **Equals 'Hindu [5]'** OR
Skip when ***rel_sa (4.12)*** **Equals 'Buddhist [6]'** OR
Skip when ***rel_sa (4.12)*** **Equals 'Christian [7]'** OR
Skip when ***rel (4.13)*** **Equals 'Catholic [1]'** OR
Skip when ***rel (4.13)*** **Equals 'Moslem [2]'** OR
Skip when ***rel (4.13)*** **Equals 'Protestant [3]'** OR
Skip when ***rel (4.13)*** **Equals 'Hindu [5]'**

4.14 relo

Specify religion

Expects a single line text response (**required**)

Prerequisites
Skip when ***country (3.1)*** **Not Equal 'India - Sehore [2]'**

4.15 caste_in

What is your caste?

Expects a single option response (**required**)

- General caste [1]
- OBC [2]
- ST [3]
- SC [4]
- Other [77]

Prerequisites
Skip when ***caste_in (4.15)*** **Equals 'General caste [1]'** OR
Skip when ***caste_in (4.15)*** **Equals 'OBC [2]'** OR
Skip when ***caste_in (4.15)*** **Equals 'ST [3]'** OR
Skip when ***caste_in (4.15)*** **Equals 'SC [4]'** OR
Skip when ***country (3.1)*** **Equals 'Ethiopia - Sodo [1]'** OR
Skip when ***country (3.1)*** **Equals 'South Africa - Dr KK [4]'** OR
Skip when ***country (3.1)*** **Equals 'Uganda - Kamuli [5]'**

4.16 casteo

Specify other caste

Expects a single line text response (**required**)

Prerequisites
Skip when ***participant (3.3)*** **Not Equal 'Caregiver [1]'**

4.17 ptname

You are part of this study because you are the caregiver for a person who is enrolled in the PRIME study. How shall I refer to this person during this interview? (e.g. Your brother, your auntie, Sujit-ji, etc).

Expects a single line text response (**required**)

Prerequisites
Skip when ***participant (3.3)*** **Not Equal 'Caregiver [1]'**

4.18 relation

What is your relationship to __{376541}__?

Expects a single option response (**required**)

- Parent [1]
- Sibling [2]
- Child [3]
- Spouse [4]
- Grandparent [5]
- Other [77]

Prerequisites
Skip when ***relation (4.18)*** **Not Equal 'Other [77]'** OR
Skip when ***participant (3.3)*** **Not Equal 'Caregiver [1]'**

4.19 relationo

Please specify the relationship to __{376541}__

Expects a single line text response (**required**)

Section Prerequisites
Skip when *visit (3.7)* **Not Equal'Baseline [1]'** OR
Skip when *participant (3.3)* **Equals'Caregiver [1]'** OR
Skip when *negmd (3.8)* **Equals'Negative [not recruited] [0]'**

Section 5. Clinical History

5.1 starty

You are part of this study due to problems with Label(**cohort (3.2)** , Q(**cohort (3.2)** ,0)) . How long ago did you MOST RECENTLY start to have these problems? [Enter YEARS ago. If less than 1 year, enter 0]

Expects a numeric response (**required**)

Prerequisites
Skip when *starty (5.1)* **Greater Than '0'**

5.2 startm

How long ago did you MOST RECENTLY start to have these problems? [Enter MONTHS ago. If less than 1 month, enter 0]

Expects a numeric response (**required**)

Constraints

*Response must be **Less Than'11'***

Prerequisites
Skip when *startm (5.2)* **Greater Than '0'** OR
Skip when *starty (5.1)* **Greater Than '0'**

5.3 startd

How long ago did you MOST RECENTLY start to have these problems? [Enter number of DAYS ago]

Expects a numeric response (**required**)

Constraints

*Response must be **Less Than'31'***

5.4 rechelp

Did you seek any help for these problems? By help I mean the kind of support you can get from someone like a traditional healer, community health worker or medical professional.

Expects a single option response (**required**)

- No [0]
- Yes [1]

Branches

If response **Equals 'No [0]'** then skip to ***endofclinicalhistory (5.23)***

5.5 rechelpy

How long ago did you first seek help for this MOST RECENT episode of Label(**cohort (3.2)** , Q(**cohort (3.2)** ,0)) ? [Answer in YEARS. If <1 year, then enter 0]

Expects a numeric response (**required**)

Prerequisites
Skip when *rechelpy (5.5)* **Greater Than '0'**

5.6 rechelpm

How long ago did you first seek help for this most recent episode? [Answer in MONTHS. If <1 month, enter 0]

Expects a numeric response (**required**)

Constraints

*Response must be **between'0' and '11'***

Prerequisites
Skip when ***rechelpm (5.6)*** **Greater Than '0'** OR
Skip when ***rechelpy (5.5)*** **Greater Than '0'**

5.7 rechelpd

How long ago did you first seek help for this most recent episode? [Enter number of DAYS]

Expects a numeric response (**required**)

Constraints

*Response must be **between'0' and '30'***

5.8 rechelpwho

From whom did you FIRST seek help for problems with Label(**cohort (3.2)** , Q(**cohort (3.2)** ,0)) ?

Expects a single option response (**required**)

- Traditional healer [1]
- Religious or spiritual advisor [2]
- Community health worker [3]
- Nurse/midwife [4]
- Pharmacist [5]
- General doctor [6]
- Specialist doctor (non-psychiatrist) [7]
- Psychiatrist [8]
- Another mental health worker [9]
- Someone else (specify) [77]

Prerequisites
Skip when ***rechelpwho (5.8)*** **Not Equal 'Someone else (specify) [77]'**

5.9 rechelpwhoo

First sought help from other (specify):

Expects a single line text response (**required**)

5.10 rechow

How much did Label(**rechelpwho (5.8)** , Q(**rechelpwho (5.8)** ,0)) help you?

Expects a single option response (**required**)

- Did not help at all [0]
- Helped a bit [1]
- Helped a lot [2]
- Don't know [888]

5.11 txseen

Have you sought help from any of the following people since the problems with Label(**cohort (3.2)** , Q(**cohort (3.2)** ,0)) most recently started?

Expects multiple selected options (**required**)

- None [0]
- Traditional healer [1]
- Religious or spiritual advisor [2]
- Community health worker [3]
- Nurse/midwife [4]
- Pharmacist [5]
- General doctor [6]
- Specialist (non-psychiatrist) doctor [7]
- Psychiatrist [8]
- Another mental health worker [9]
- Others (specify) [77]

Prerequisites
Skip when *txseen (5.11)* **Excludes 'Others (specify) [77]'**

5.12 txseeno

Also sought help from other (specify):

Expects a single line text response (**required**)

Prerequisites
Skip when *txseen (5.11)* **Excludes 'Traditional healer [1]'**

5.13 txtrad_help

How much did the TRADITIONAL HEALER help you?

Expects a single option response (**required**)

- Did not help at all [0]
- Helped a bit [1]
- Helped a lot [2]
- Don't know [888]

Prerequisites
Skip when *txseen (5.11)* **Excludes 'Religious or spiritual advisor [2]'**

5.14 txrel_help

How much did the RELIGIOUS OR SPIRITUAL ADVISOR help you?

Expects a single option response (**required**)

- Did not help at all [0]
- Helped a bit [1]
- Helped a lot [2]
- Don't know [888]

Prerequisites
Skip when *txseen (5.11)* **Excludes 'Community health worker [3]'**

5.15 txchw_help

How much did the COMMUNITY HEALTH WORKER help you?

Expects a single option response (**required**)

- Did not help at all [0]
- Helped a bit [1]
- Helped a lot [2]
- Don't know [888]

Prerequisites
Skip when *txseen (5.11)* **Excludes 'Nurse / midwife [4]'**

5.16 txnur_help

How much did the NURSE / MIDWIFE help you?

Expects a single option response (**required**)

- Did not help at all [0]
- Helped a bit [1]
- Helped a lot [2]
- Don't know [888]

Prerequisites
Skip when *txseen (5.11)* **Excludes 'Pharmacist [5]'**

5.17 txpharm_help

How much did the PHARMACIST help you?

Expects a single option response (**required**)

- Did not help at all [0]
- Helped a bit [1]
- Helped a lot [2]
- Don't know [888]

Prerequisites
Skip when *txseen (5.11)* **Excludes 'General doctor [6]'**

5.18 txgen_help

How much did the GENERAL DOCTOR help you?

Expects a single option response (**required**)

- Did not help at all [0]
- Helped a bit [1]
- Helped a lot [2]
- Don't know [888]

Prerequisites
Skip when *txseen (5.11)* **Excludes 'Specialist (non-psychiatrist) doctor [7]'**

5.19 txspec_help

How much did the SPECIALIST DOCTOR help you?

Expects a single option response (**required**)

- Did not help at all [0]
- Helped a bit [1]
- Helped a lot [2]
- Don't know [888]

Prerequisites
Skip when *txseen (5.11)* **Excludes 'Psychiatrist [8]'**

5.20 txpsy_help

How much did the PSYCHIATRIST help you?

Expects a single option response (**required**)

- Did not help at all [0]
- Helped a bit [1]
- Helped a lot [2]
- Don't know [888]

Prerequisites
Skip when *txseen (5.11)* **Excludes 'Another mental health worker [9]'**

5.21 txoment_help

How much did the OTHER MENTAL HEALTH WORKER help you?

Expects a single option response (**required**)

- Did not help at all [0]
- Helped a bit [1]
- Helped a lot [2]
- Don't know [888]

Prerequisites
Skip when *txseen (5.11)* **Excludes 'Others (specify) [77]'**

5.22 txo_help

How much did the Q**txseeno (5.12)** help you?

Expects a single option response (**required**)

- Did not help at all [0]
- Helped a bit [1]
- Helped a lot [2]
- Don't know [888]

5.23 endofclinicalhistory

[End of section]

Section Prerequisites
Skip when *cohort (3.2)* **Not Equal'psychosis [4]'** OR
Skip when *country (3.1)* **Equals'Ethiopia - Sodo [1]'** OR
Skip when *country (3.1)* **Equals'India - Sehore [2]'** OR
Skip when *country (3.1)* **Equals'Nepal - Chitwan [3]'** OR
Skip when *country (3.1)* **Equals'South Africa - Dr KK [4]'** OR
Skip when *country (3.1)* **Equals'Uganda - Kamuli [5]'**

Section 6. PANSS

6.1 PANSS1

In the past 3 months, did you (or your relative) feel afraid of people for no reason, such as thinking that food is poisoned or someone wants to harm you?

Expects a single option response (**required**)

- Never [0]
- Once [1]
- Few times [2]
- Often [3]
- Continupusly [4]

6.2 PANSS2

In the past 3 months, did you (or your relative) speak about things and ideas that that other people didnt understand?

Expects a single option response (**required**)

- Never [0]
- Once [1]
- Few times [2]
- Often [3]
- Continuously [4]

6.3 PANSS3

In the past 3 months, did you (or your relative) hear voices that other people did not hear or voices telling you to do things even when other people werent around or did you see things that other people said they could not see?

Expects a single option response (**required**)

- Never [0]
- Once [1]
- A few times [2]
- Often [3]
- Continuously [4]

6.4 PANSS4

In the past 3 months, have you felt happy and energetic for no reason?

Expects a single option response (**required**)

- Never [0]
- A few times [2]
- Once [1]
- Often [3]
- Continuously [4]

6.5 PANSS5

In the past 3 months, have you (your relative) been acting as if you have special powers/abilities, or are a great person in society?

Expects a single option response (**required**)

- Never [0]
- Once [1]
- A few times [2]
- Often [3]
- Continuously [4]

6.6 PANSS6

In the past 3 months, have you (your relative) felt that people want to follow and hurt you, but other people do not believe you?

Expects a single option response (**required**)

- Never [0]
- Once [1]
- A few times [2]
- Often [3]
- Continuously [4]

6.7 PANSS7

In the past 3 months, have you (your relative) felt angry often, had thoughts of wanting to hurt people, or threatened other people with violence?

Expects a single option response (**required**)

- Never [0]
- Once [1]
- A few times [2]
- Often [3]
- Continuously [4]

6.8 PANNS8

In the past 3 months, have you (your relative) heard from other people that you look distant, unengaged?

Expects a single option response (**required**)

- Never [0]
- Once [1]
- A few times [2]
- Often [3]
- Continuously [4]

6.9 PANSS9

In the past 3 months, have you (your relative) felt like you didnt feel the same way as people around you (e.g. you werent happy at times when they were happy, or you werent sad at times when they were sad?

Expects a single option response (**required**)

- Never [0]
- Once [1]
- A few times [2]
- Often [3]
- Continuously [4]

6.10 PANSS10

In the past 3 months, have you (your relative) found it difficult to make friends and maintain old friendship OR do you find it difficult to make small talk with people?

Expects a single option response (**required**)

- Never [0]
- Once [1]
- A few times [2]
- Often [3]
- Continuously [4]

6.11 PANSS11

In the past 3 months, have you (your relative) had a lack of interest in doing things with other people?

Expects a single option response (**required**)

- Never [0]
- Once [1]
- A few times [2]
- Often [3]
- Continuously [4]

6.12 PANSS12

In the past 3 months, have you (your relative) had difficulty understanding familiar sayings, or learning new ideas?

Expects a single option response (**required**)

- Never [0]
- Once [1]
- A few times [A few times]
- Often [3]
- Continuously [4]

6.13 PANSS13

In the past 3 months, have you (your relative) been speaking rarely or responded with only yes,no,I dont know to questions?

Expects a single option response (**required**)

- Never [0]
- Once [1]
- A few times [2]
- Often [3]
- Continuously [4]

6.14 PANSS14

In the past 3 months, have you (your family member) been thinking and talking about the same over and over?

Expects a single option response (**required**)

- Never [0]
- Once [1]
- A few times [2]
- Often [3]
- Continuously [4]

Section Prerequisites
Skip when ***participant (3.3)*** **Equals'Caregiver [1]'** OR
Skip when ***negmd (3.8)*** **Equals'Negative [not recruited] [0]'** OR
Skip when ***diagconfirmed (3.9)*** **Equals'No [0]'**

Section 7. AUDIT

Prerequisites
Skip when ***country (3.1)*** **Equals 'South Africa - Dr KK [4]'**

7.1 AUDIT

In this section we will ask questions about drinking alcohol. By alcohol I mean drinks like....

Prerequisites
Skip when ***country (3.1)*** **Not Equal 'South Africa - Dr KK [4]'**

7.2 AUDIT_SA1

In this section we will ask questions about drinking alcohol. By alcohol I mean drinks like... [show alcohol chart here- FLASH CARD 1]

Prerequisites
Skip when ***cohort (3.2)*** **Equals 'alcohol [3]'**

7.3 AUDEVER

Have you ever had a drink containing alcohol?

Expects a single option response (**required**)

- No, I have never consumed alcohol [0]
- Yes, I have consumed alcohol [1]

Branches

If response **Equals 'No, I have never consumed alcohol [0]'** then skip to ***endofaudit (7.18)***

7.4 AUDYEAR

Now I am going to ask you some questions about your use of alcoholic beverages during the past THREE months. That is, your use of alcohol beverages since __{377429}__.

Prerequisites
Skip when ***cohort (3.2)*** **Equals 'alcohol [3]'**

7.5 AUD3MONTH

Have you had a drink containing alcohol in the past three months?

Expects a single option response (**required**)

- No, I have not consumed alcohol [0]
- Yes, I have consumed alcohol [1]

Branches

If response **Equals 'No, I have not consumed alcohol [0]'** then skip to ***endofaudit (7.18)***

7.6 AUD1

How often do you have a drink containing alcohol?

Expects a single option response (**required**)

- Never [0]
- Monthly or less [1]
- 2-4 times a month [2]
- 2-3 times a week [3]
- 4 or more times a week [4]

7.7 AUD2

How many drinks containing alcohol do you have on a typical day when you are drinking?

Expects a single option response (**required**)

- 1-2 [0]
- 3-4 [1]
- 5-6 [2]
- 7-9 [3]
- 10 or more [4]

Prerequisites
Skip when *country (3.1)* **Not Equal 'South Africa - Dr KK [4]'**

7.8 AUD_SA2

[show FLASH CARD 2]

7.9 AUD3

How often do you have six or more drinks on one occasion?

Expects a single option response (**required**)

- Never [0]
- Less than monthly [1]
- Monthly [2]
- Weekly [3]
- Daily or almost daily [4]

7.10 Set Total of AUD2 and AUD3

Operator

This field is not displayed on the handset, Operator: Set(**Total of AUD2 and AUD3 (1.2)** , Sum of (Q 37416) , Q **AUD3 (7.9)**))

Prerequisites
Skip when *Total of AUD2 and AUD3 (1.2)* **Equals '0'**

7.11 AUD4

How often during the last 3 months have you found that you were not able to stop drinking once you had started?

Expects a single option response (**required**)

- Never [0]
- Less than monthly [1]
- Monthly [2]
- Weekly [3]
- Daily or almost daily [4]

Prerequisites
Skip when *Total of AUD2 and AUD3 (1.2)* **Equals '0'**

7.12 AUD5

How often during the last 3 months have you failed to do what was normally expected from you because of drinking?

Expects a single option response (**required**)

- Never [0]
- Less than monthly [1]
- Monthly [2]
- Weekly [3]
- Daily or almost daily [4]

Prerequisites
Skip when *Total of AUD2 and AUD3 (1.2)* **Equals '0'**

7.13 AUD6

How often during the last 3 months have you needed a first drink in the morning to get yourself going after a heavy drinking session?

Expects a single option response (**required**)

- Never [0]
- Less than monthly [1]
- Monthly [2]
- Weekly [3]
- Daily or almost daily [4]

Prerequisites
Skip when ***Total of AUD2 and AUD3 (1.2)*** **Equals '0'**

7.14 AUD7

How often during the last 3 months have you had a feeling of guilt or remorse after drinking?

Expects a single option response (**required**)

- Never [0]
- Less than monthly [1]
- Monthly [2]
- Weekly [3]
- Daily or almost daily [4]

Prerequisites
Skip when ***Total of AUD2 and AUD3 (1.2)*** **Equals '0'**

7.15 AUD8

How often during the last 3 months have you been unable to remember what happened the night before because you had been drinking?

Expects a single option response (**required**)

- Never [0]
- Less than monthly [1]
- Monthly [2]
- Weekly [3]
- Daily or almost daily [4]

7.16 AUD9

Have you or someone else been injured as a result of your drinking?

Expects a single option response (**required**)

- No [0]
- Yes, but not in the last year [2]
- Yes, during the last year [4]

7.17 AUD10

Has a relative or friend or a doctor or another health worker been concerned about your drinking or suggested you cut down?

Expects a single option response (**required**)

- No [0]
- Yes, but not in the last year [2]
- Yes, during the last year [4]

7.18 endofaudit

[End of AUDIT section]

Section Prerequisites
Skip when *cohort (3.2)* Not Equal'alcohol [3]'

Section 8. SIP2R

8.1 sip Instructions

Here are a number of events that people sometimes experience. Tell me about the frequency of these events in the past THREE months. That is, since __{377429}__.

8.2 sip1

I have been unhappy because of my drinking.

Expects a single option response (**required**)

- Never [0]
- One or a few times [1]
- Once or twice a week [2]
- Daily or almost daily [3]

8.3 sip2

Because of my drinking, I have not eaten properly.

Expects a single option response (**required**)

- Never [0]
- One or a few times [1]
- Once or twice a week [2]
- Daily or almost daily [3]

8.4 sip3

I have failed to do what is expected of me because of my drinking.

Expects a single option response (**required**)

- Never [0]
- One or a few times [1]
- Once or twice a week [2]
- Daily or almost daily [3]

8.5 sip4

I have felt guilty or ashamed because of my drinking.

Expects a single option response (**required**)

- Never [0]
- One or a few times [1]
- Once or twice a week [2]
- Daily or almost daily [3]

8.6 sip5

I have taken foolish risks when I have been drinking.

Expects a single option response (**required**)

- Never [0]
- One or a few times [1]
- Once or twice a week [2]
- Daily or almost daily [3]

8.7 sip6

When drinking, I have done impulsive things that I regretted later.

Expects a single option response (**required**)

- Never [0]
- One or a few times [1]
- Once or twice a week [2]
- Daily or almost daily [3]

8.8 sip Instructions 2

Now answer these questions about things that may have happened to you. During the past 3 months, how much has this happened?

8.9 sip7

My physical health has been harmed by my drinking.

Expects a single option response (**required**)

- Never [0]
- One or a few times [1]
- Once or twice a week [2]
- Daily or almost daily [3]

8.10 sip8

I have had money problems because of my drinking.

Expects a single option response (**required**)

- Never [0]
- One or a few times [1]
- Once or twice a week [2]
- Daily or almost daily [3]

8.11 sip9

My physical appearance has been harmed by my drinking.

Expects a single option response (**required**)

- Never [0]
- One or a few times [1]
- Once or twice a week [2]
- Daily or almost daily [3]

8.12 sip10

My family has been hurt by my drinking.

Expects a single option response (**required**)

- Never [0]
- One or a few times [1]
- Once or twice a week [2]
- Daily or almost daily [3]

8.13 sip11

A friendship or close relationship has been damaged by my drinking.

Expects a single option response (**required**)

- Never [0]
- One or a few times [1]
- Once or twice a week [2]
- Daily or almost daily [3]

8.14 sip12

My drinking has gotten in the way of my growth as a person.

Expects a single option response (**required**)

- Never [0]
- One or a few times [1]
- Once or twice a week [2]
- Daily or almost daily [3]

8.15 sip13

My drinking has damaged my social life, popularity, or reputation.

Expects a single option response (**required**)

- Never [0]
- One or a few times [1]
- Once or twice a week [2]
- Daily or almost daily [3]

8.16 sip14

I have spent too much or lost a lot of money because of my drinking.

Expects a single option response (**required**)

- Never [0]
- One or a few times [1]
- Once or twice a week [2]
- Daily or almost daily [3]

8.17 sip Instructions 3

Has this happened to you DURING THE PAST 3 MONTHS?

8.18 sip15

I have had an accident while drinking or intoxicated.

Expects a single option response (**required**)

- Never [0]
- One or a few times [1]
- Once or twice a week [2]
- Daily or almost daily [3]

Section 9. PHQ9

Prerequisites
Skip when *country (3.1)* Equals 'South Africa - Dr KK [4]'

9.1 PHQ-9 Instruction

Now I am asking you some of the problems that may have experienced in the past TWO weeks (that is, since __{393665}__). Please tell me how often have you been bothered by the following problems.

Prerequisites
Skip when *country (3.1)* Not Equal 'South Africa - Dr KK [4]'

9.2 PHQ-9 Instruction_SA

Now I am asking you some of the problems that may have experienced in the past TWO weeks (that is, since __{393665}__). [show FLASH CARD 3] Please tell me how often have you been bothered by the following problems.

9.3 PHQ1

Little interest or pleasure in doing things.

Expects a single option response (**required**)

- Not at all [0]
- Several days [1]
- More than half the days [2]
- Nearly every day [3]

9.4 PHQ2

Feeling down, depressed, or hopeless.

Expects a single option response (**required**)

- Not at all [0]
- Several days [1]
- More than half the days [2]
- Nearly every day [3]

9.5 PHQ3

Trouble falling/staying asleep, sleeping too much.

Expects a single option response (**required**)

- Not at all [0]
- Several days [1]
- More than half the days [2]
- Nearly every day [3]

9.6 PHQ4

Feeling tired or having little energy.

Expects a single option response (**required**)

- Not at all [0]
- Several days [1]
- More than half the days [2]
- Nearly every day [3]

9.7 PHQ5

Poor appetite or overeating.

Expects a single option response (**required**)

- Not at all [0]
- Several days [1]
- More than half the days [2]
- Nearly every day [3]

9.8 PHQ6

Feeling bad about yourself or that you are a failure or have let yourself or your family down.

Expects a single option response (**required**)

- Not at all [0]
- Several days [1]
- More than half the days [2]
- Nearly every day [3]

9.9 PHQ7

Trouble concentrating on things, such as reading the newspaper or watching television.

Expects a single option response (**required**)

- Not at all [0]
- Several days [1]
- More than half the days [2]
- Nearly every day [3]

9.10 PHQ8

Moving or speaking so slowly that other people could have noticed. Or the opposite being so fidgety or restless that you have been moving around a lot more than usual.

Expects a single option response (**required**)

- Not at all [0]
- Several days [1]
- More than half the days [2]
- Nearly every day [3]

9.11 PHQ9

Thoughts that you would be better off dead or of hurting yourself in some way.

Expects a single option response (**required**)

- Not at all [0]
- Several days [1]
- More than half the days [2]
- Nearly every day [3]

9.12 PHQ10

How difficult have these problems made it for you to do your work, take care of things at home, or get along with other people?

Expects a single option response (**required**)

- Not difficult at all [0]
- Somewhat difficult [1]
- Very difficult [2]
- Extremely difficult [3]

9.13 Set PHQ total

Operator

This field is not displayed on the handset, Operator: Set(**TotalPHQ (1.18)** , Sum of (q37418) , q374189 , q374190 , q374191 , q374192 , q374193 , q374194 , q374195 , q374196))

Prerequisites
Skip when ***negmd (3.8)*** **Not Equal 'Negative [not recruited] [0]'** OR
Skip when ***diagconfirmed (3.9)*** **Equals 'Yes [1]'** OR
Skip when ***cohort (3.2)*** **Not Equal 'maternal depression [2]'**

9.14 phq9end

[End of PHQ-9 section]

Expects a single option response (**required**)

- Click 'Next' to end interview [1]

Branches

If response **Equals 'Click 'Next' to end interview [1]'** then skip to ***comments (47.3)***

Section Prerequisites
Skip when ***participant (3.3)*** **Equals'Caregiver [1]'**

Section 10. Suicidality

10.1 suicdalityinstruction

The next questions are about feelings and events that may have occurred in the past THREE months. That is, since __{377429}__.

10.2 SUITHINK

Have you thought of taking your life in the past 3 months?

Expects a single option response (**required**)

- No [0]
- Yes [1]

Branches

If response **Equals 'No [0]'** then skip to ***endofsuicidality (10.11)***

10.3 SUIPLAN

Did you make a plan for taking your own life at any time in the past 3 months?

Expects a single option response (**required**)

- No [0]
- Yes [1]

Branches

If response **Equals 'No [0]'** then skip to ***endofsuicidality (10.11)***

10.4 SUIATT

Have you attempted to take your own life in the past 3 months?

Expects a single option response (**required**)

- No [0]
- Yes [1]

Branches

If response **Equals 'No [0]'** then skip to ***endofsuicidality (10.11)***

10.5 SUIMED

Did it require medical attention?

Expects a single option response (**required**)

- No [0]
- Yes [1]

10.6 SUIDISC

In the past 3 months, have you spoken to anyone about thinking about or attempting to take your own life?

Expects a single option response (**required**)

- No [0]
- Yes [1]

Branches

If response **Equals 'No [0]'** then skip to ***SUITX (10.9)***

10.7 suidisc_

To whom have you spoken?

Expects multiple selected options (**required**)

- Friend / neighbor [1]
- Spouse/partner [2]
- Other family member [3]
- Employer/coworker [4]
- Traditional healer [5]
- Health care worker (e.g. nurse/doctor, specialist) [6]
- Religious or spiritual healer [7]
- Other [77]

Prerequisites
Skip when ***suidisc_ (10.7)*** **Excludes 'Other [77]'**

10.8 SUIDISCO

Please specify:

Expects a single line text response (**required**)

10.9 SUITX

Did you receive any treatment for thinking about or attempting to take your own life?

Expects a single option response (**required**)

- No [0]
- Yes [1]
- Don't Know [888]

Branches

If response **Equals 'No [0]'** then skip to ***endofsuicidality (10.11)***

10.10 SUITXO

What treatment did you receive?

Expects a single line text response (**required**)

10.11 endofsuicidality

[End of section]

Section Prerequisites
Skip when ***cohort (3.2)*** **Not Equal'epilepsy [5]'** OR
Skip when ***participant (3.3)*** **Equals'Caregiver [1]'**

Section 11. Epilepsy Severity

11.1 epilasty

How long ago did you last experience a seizure? [Enter number of YEARS ago. If <2 years, then enter 0]

Expects a numeric response (**required**)

Constraints

*Response must be **Not Equal'1'***

Prerequisites
Skip when ***epilasty (11.1)*** **Greater Than '1'**

11.2 epilastm

How long ago did you last experience a seizure? [Enter 0-23 MONTHS ago. If <1 month, enter 0]

Expects a numeric response (**required**)

Constraints

*Response must be **between'0' and '23'***

Prerequisites
Skip when ***epilastm (11.2)*** **Greater Than '0'**

11.3 epilastd

How long ago did you last experience a seizure? [Enter number of DAYS ago]

Expects a numeric response (**required**)

Constraints

*Response must be **between'0' and '31'***

11.4 epinum

How many seizures did you have in the past 30 days? That is, since __{377317}__.

Expects a numeric response (**required**)

Section Prerequisites
Skip when *cohort (3.2)* **Not Equal'maternal depression [2]'**

Section 12. Maternal Health

12.1 pregstatus

[Participant's pregnancy status]

Expects a single option response (**required**)

- Antenatal [1]
- Postnatal [2]

Branches

If response **Equals 'Postnatal [2]'** then skip to ***postnatal (12.10)***

12.2 antenatal

The next few questions about your experience in antenatal care.

12.3 monpreg

How many months pregnant are you? [Use 88 for don't know]

Expects a numeric response (**required**)

Constraints

*Response must be **between'0' and '9'** OR*

*Response must be **Equals'88'***

12.4 eddknown

Do you know approximately when your due date is?

Expects a single option response (**required**)

- No [0]
- Yes [1]

Prerequisites
Skip when *eddknown (12.4)* **Equals 'No [0]'**

12.5 edd

When is your approximate due date?

Expects a date response (**required**)

12.6 antecare

How many times have you received antenatal care during this pregnancy?

Expects a numeric response (**required**)

12.7 tetinj

During this pregnancy, were you given an injection in the arm to prevent the baby from getting tetanus, that is, convulsions (infections) after birth?

Expects a single option response (**required**)

- No [0]
- Yes [1]
- Don't know [88]

Prerequisites
Skip when *tetinj (12.7)* **Not Equal 'Yes [1]'**

12.8 tetinjnum

During this pregnancy, how many times did you get this tetanus injection?

Expects a numeric response (**required**)

12.9 gotoend

Operator

This field is not displayed on the handset, Operator: GoTo(**endofmaternal (12.18)**)

12.10 postnatal

The next few questions about your experience with postnatal care.

12.11 pregendm

How long ago did your pregnancy end? [Enter in MONTHS ago. If <2 months ago, enter 0]

Expects a numeric response (**required**)

Constraints

*Response must be **Not Equal'1'***

Prerequisites
Skip when ***pregendm (12.11)*** **Not Equal '0'**

12.12 pregendw

How long ago did your pregnancy end? [Enter in WEEKS ago. If <2 weeks ago, enter 0]

Expects a numeric response (**required**)

Constraints

*Response must be **Not Equal'1'***

Prerequisites
Skip when ***pregendw (12.12)*** **Not Equal '0'** OR
Skip when ***pregendm (12.11)*** **Not Equal '0'**

12.13 pregendd

How long ago did your pregnancy end? [Enter DAYS ago]

Expects a numeric response (**required**)

Constraints

*Response must be **between'1' and '14'***

12.14 livebirth

[Assess how the pregnancy ended]

Expects a single option response (**required**)

- Abortion/miscarriage/stillbirth/infant mortality [0]
- Live birth [1]

Branches

If response **Equals 'Abortion/miscarriage/stillbirth/infant mortality [0]'** then skip to ***endofmaternal (12.18)***

12.15 breastfeed

Are you still breastfeeding?

Expects a single option response (**required**)

- No [0]
- Yes [1]

12.16 vacccard

Do you have a card where your child's vaccination are written down? May I see it please?

Expects a single option response (**required**)

- Yes, and can see card [2]
- Yes, but can't see card [1]
- No card [0]

Prerequisites
Skip when ***vacccard (12.16)*** **Equals 'No card [0]'**

12.17 vacctype

Please tell me if your child received any of the following vaccinations: [Use vaccination card if available] [Choose all that apply]

Expects multiple selected options (**required**)

- ☐ A BCG vaccination against tuberculosis, that is, an injection in the right upper arm that usually causes a scar [1]
- ☐ Polio vaccine, that is, drops in the mouth [2]
- ☐ A DPT vaccination, that is, an injection given in the upper thigh sometimes at the same time as polio drops [3]
- ☐ A measles injection or an MMR injection, that is, a shot in the left upper arm [at the age of 9 months or older] to prevent him/her from getting measles [4]

12.18 endofmaternal

[End of section]

Section Prerequisites
Skip when *participant (3.3)* Equals'Caregiver [1]'

Section 13. WHODAS

13.1 WHO Disability Assessment Instruction 1

The next part of this interview is about difficulties people have because of health conditions. [Fieldworker: Hand flashcard to respondent] By health condition I mean diseases or illnesses, other health problems that may be short or long lasting, injuries, mental or emotional problems and problems with alcohol or drugs.

Prerequisites
Skip when *country (3.1)* Equals 'South Africa - Dr KK [4]'

13.2 WHO Disability Assessment Instruction 2

I remind you to keep all of your health problems in mind as you answer the questions. When I ask you about difficulties in doing an activity think about: [Point to flashcard #1]. Increased effort Discomfort or pain Slowness Changes in the way you do the activity

---

Prerequisites
Skip when *country (3.1)* Not Equal 'South Africa - Dr KK [4]'

13.3 WHO Disability Assessment Instruction 2_SA

I remind you to keep all of your health problems in mind as you answer the questions. When I ask you about difficulties in doing an activity think about: [Show FLASH CARD 4]. Increased effort Discomfort or pain Slowness Changes in the way you do the activity

---

13.4 WHO Disability Assessment Instruction 3

When answering, Id like you to think back over the last 30 days (since __{377317}__). I also would like you to answer these questions thinking about how much difficulty you have, on average over the past 30 days, while doing the activity as you usually do it.

13.5 WHO Disability Assessment Instruction 4

[Hand flashcard #2 to interviewee] Use this scale when responding. [Read scale aloud]: None, mild, moderate, severe, extreme or cannot do. [Fieldworker: Note, Flashcards #1 and #2 should remain visible to the respondent throughout the interview]

13.6 OVERALL

How do you rate your overall health in the past 30 days?

Expects a single option response (**required**)

- Very good [1]
- Good [2]
- Moderate [3]
- Bad [4]
- Very bad [5]

Prerequisites
Skip when *country (3.1)* Equals 'South Africa - Dr KK [4]'

13.7 WHO Disability Assessment Instruction 8

[Fieldworker: Show flashcard #2 to participant.] In the last 30 days how much difficulty did you have in:

Prerequisites
Skip when *country (3.1)* Not Equal 'South Africa - Dr KK [4]'

13.8 WHO Disability Assessment Instruction 8_SA

[Show FLASH CARD 5] In the last 30 days how much difficulty did you have in:

13.9 STAND

Standing for long periods such as 30 minutes?

Expects a single option response (**required**)

- None [1]
- Mild [2]
- Moderate [3]
- Severe [4]
- Extreme/cannot do [5]

13.10 HOUSE

Taking care of your household responsibilities?

Expects a single option response (**required**)

- None [1]
- Mild [2]
- Moderate [3]
- Severe [4]
- Extreme/cannot do [5]

13.11 LEARN

Learning a new task, for example, learning how to get to a new place?

Expects a single option response (**required**)

- None [1]
- Mild [2]
- Moderate [3]
- Severe [4]
- Extreme/cannot do [5]

13.12 JOIN

How much of a problem did you have in joining community activities (for example, festivities, religious or other activities) in the same way as anyone else can?

Expects a single option response (**required**)

- None [1]
- Mild [2]
- Moderate [3]
- Severe [4]
- Extreme/cannot do [5]

13.13 EMOTE

How much have you been emotionally affected by your health problems?

Expects a single option response (**required**)

- None [1]
- Mild [2]
- Moderate [3]
- Severe [4]
- Extreme/cannot do [5]

13.14 WHO Disability Assessment Instruction 9

In the last 30 days, how much difficulty did you have in:

13.15 CONC

Concentrating on doing something for 10 minutes?

Expects a single option response (**required**)

- None [1]
- Mild [2]
- Moderate [3]
- Severe [4]
- Extreme/cannot do [5]

13.16 WALK

Walking a long distance such as a kilometer?

Expects a single option response (**required**)

- None [1]
- Mild [2]
- Moderate [3]
- Severe [4]
- Extreme/cannot do [5]

13.17 WASH

Washing your whole body?

Expects a single option response (**required**)

- None [1]
- Mild [2]
- Moderate [3]
- Severe [4]
- Extreme/cannot do [5]

13.18 DRESS

Getting dressed?

Expects a single option response (**required**)

- None [1]
- Mild [2]
- Moderate [3]
- Severe [4]
- Extreme/cannot do [5]

13.19 DEAL

Dealing with people you do not know?

Expects a single option response (**required**)

- None [1]
- Mild [2]
- Moderate [3]
- Severe [4]
- Extreme/cannot do [5]

13.20 FRIEND

Maintaining a friendship?

Expects a single option response (**required**)

- None [1]
- Mild [2]
- Moderate [3]
- Severe [4]
- Extreme/cannot do [5]

13.21 DAY

Your day to day work?

Expects a single option response (**required**)

- None [1]
- Mild [2]
- Moderate [3]
- Severe [4]
- Extreme/cannot do [5]

13.22 **INTERF**

Overall, how much did these difficulties interfere with your life?

Expects a single option response (**required**)

- None [1]
- Mild [2]
- Moderate [3]
- Severe [4]
- Extreme/cannot do [5]

13.23 **DIFFDAYS**

Overall, in the past 30 days, how many days were these difficulties present?

Expects a numeric response (**required**)

Constraints

*Response must be **Less Than or Equal'30'***

13.24 **UNABLE**

In the past 30 days, for how many days were you totally unable to carry out your usual activities or work because of any health condition?

Expects a numeric response (**required**)

Constraints

*Response must be **Less Than or Equal'30'***

13.25 **CUTBACK**

In the past 30 days, not counting the days you were totally unable, for how many days did you cut back or reduce your usual activities or work because of any health condition?

Expects a numeric response (**required**)

Constraints

*Response must be **Less Than or Equal'30'***

Section Prerequisites
Skip when ***Participant-proxy (1.4)*** **Equals'3'**

Section 14. Inpatient care

Prerequisites
Skip when ***country (3.1)*** **Not Equal 'Uganda - Kamuli [5]'**

14.1 ipci

[These question must be answered by the caregiver on behalf of the patient - the following questions refer to the patient's health care.]

14.2 Inpatientcareinstruction

I would now like to know about your recent experiences with obtaining health care. Following questions will be about your inpatient care which you have taken in the last year

---

14.3 ipd12mo

In the last year, have you ever stayed overnight in a hospital? That is, since __{377431}__.

Expects a single option response (**required**)

- No [0]
- Yes [1]

Branches

If response **Equals 'No [0]'** then skip to ***outpatient instruction (16.2)***

14.4 ipd12num

How many times have you been admitted into a hospital in the last year?

Expects a numeric response (**required**)

14.5 ipd3num

How many of those visits occurred in the past 3 months? That is, since __{377429}__.

Expects a numeric response (**required**)

Constraints

*Response must be **Less Than or Equal'Qipd12num (14.4)'***

Repeat this section for value of ***ipd12num (14.4)***

Section Prerequisites
Skip when ***Participant-proxy (1.4)*** **Equals'3'**

Section 15. Inpatient care repeat

15.1 IN Instruction 1

For each separate hospital admission you have had, please complete the following: [Start with the MOST RECENT hospital admissions]

15.2 ipdwhy

For your __{LABEL(375641,REPEAT IDX admission: Why were you admitted?

Expects a single option response (**required**)

- infectious disease (e.g. malaria) [1]
- maternal / perinatal condition [2]
- acute condition (e.g. flu, cough) [3]
- injury [4]
- sleep problems [5]
- depression or anxiety [6]
- alcohol problems [7]
- other mental health problems [8]
- other chronic disease (e.g. heart, diabetes) [9]
- other condition [77]
- don't know [888]

Prerequisites
Skip when ***ipdwhy (15.2)*** **Not Equal 'other condition [77]'**

15.3 ipdwhyo

Why were you admitted? Please specify:

Expects a single line text response (**required**)

15.4 ipdwhen

When were you admitted for Label(**ipdwhy (15.2)** , Q(**ipdwhy (15.2)** ,0)) ? [If the exact date is not known, prompt the participant for an approximate date: What month was it? Was it the beginning, middle or end of the month?]

Expects a date response (**required**)

Constraints

Response must be ***Greater Than or Equal'__{377431}__'***

15.5 ipdwhere

Where were you admitted for Label(**ipdwhy (15.2)** , Q(**ipdwhy (15.2)** ,0)) ?

Expects a single option response (**required**)

- Charity / church-run hospital [1]
- Private hospital [2]
- Government hospital [3]
- Other [77]

Prerequisites
Skip when ***ipdwhere (15.5)*** **Not Equal 'Other [77]'**

15.6 ipdwhereo

Please specify the location of the hospital admission

Expects a single line text response (**required**)

15.7 ipddays

How long did you stay in the Label(**ipdwhere (15.5)** , Q(**ipdwhere (15.5)** ,0)) for Label(**ipdwhy (15.2)** , Q(**ipdwhy (15.2)** ,0)) ? [Enter number of DAYS]

Expects a numeric response (**required**)

15.8 ipdcost

How much did you, your family or friends have to pay to the Label(**ipdwhere (15.5)** , Q(**ipdwhere (15.5)** ,0)) (i.e. for hospital fees, medicines, investigations) when you were admitted for Label(**ipdwhy (15.2)** , Q(**ipdwhy (15.2)** ,0)) ? [Enter fees in __{LABEL(375774,__{374020}__)}__ units]

Expects a numeric response (**required**)

Section Prerequisites
Skip when ***Participant-proxy (1.4)*** **Equals'3'**

Section 16. Outpatient care

Prerequisites
Skip when ***country (3.1)*** **Not Equal 'Uganda - Kamuli [5]'**

16.1 opci

[These question must be answered by the caregiver on behalf of the patient - the following questions refer to the patient's health care.]

16.2 outpatient instruction

Excluding those times you may have been admitted for inpatient care, I want to know how many times you met any of the following health care providers in the past THREE months. That is, since __{377429}__.

16.3 tradno

Traditional healer/spiritualist/ herbalist

Expects a numeric response (**required**)

16.4 chwno

Community health worker

Expects a numeric response (**required**)

16.5 nursno

Nurse or midwife

Expects a numeric response (**required**)

16.6 pharmno

Pharmacist

Expects a numeric response (**required**)

16.7 gendocno

General medical doctor

Expects a numeric response (**required**)

16.8 specdocno

Specialist (non-psychiatric) medical doctor

Expects a numeric response (**required**)

16.9 psyno

Psychiatrist

Expects a numeric response (**required**)

16.10 counsno

Counsellor

Expects a numeric response (**required**)

16.11 psynurseno

Psychiatric nurse

Expects a numeric response (**required**)

16.12 swno

Social worker

Expects a numeric response (**required**)

16.13 relno

Religious or spiritual advisor

Expects a numeric response (**required**)

16.14 omhno

Any other mental health professionals (e.g. non-counselor, non-psychiatrist, non-psychiatric nurse)

Expects a numeric response (**required**)

Prerequisites
Skip when ***omhno (16.14)*** **Equals '0'**

16.15 omhnoo

Please specify the other mental health provider:

Expects a single line text response (**required**)

16.16 Set Health Providers

Operator

This field is not displayed on the handset, Operator: __{SET(374182, Sum of (__{37483}),__{374834}__,__{374835}__,__{374836}__,__{374837}__,__{374838}__,__{374839}__,__{374840}__,__{374841}__,__{374842}__,__{374843}__,__{374844}__)}__)}__

16.17 Reprocess Provider Sum

Operator

This field is not displayed on the handset, Operator: Reprocess(**Number Health Providers (1.3)**)

16.18 OUT TOT

In the last 3 months you have visited health providers __{374182}__ time(s). I will now ask you questions about each of these visits. We will start with your MOST RECENT visit.

Repeat this section for value of ***Number Health Providers (1.3)***

Section Prerequisites
Skip when ***Participant-proxy (1.4)*** **Equals'3'**

Section 17. Outpatient Care Repeat

17.1 opdwho

For your __{LABEL(375641,REPEAT IDX outpatient visit, who did you see?

Expects a single option response (**required**)

- Traditional healer/spiritualist/herbalist [1]
- Community health worker [2]
- Nurse / midwife [3]
- Pharmacist [4]
- General doctor [5]
- Specialist doctor [6]
- Psychiatrist [7]
- Counsellor [8]
- Psychiatric nurse [9]
- Social Worker [10]
- Religious or spiritual advisor [11]
- Other [77]
- Don't know [888]

Prerequisites
Skip when ***opdwho (17.1)*** **Not Equal 'Other [77]'**

17.2 opdwhoo

Please specify the provider:

Expects a single line text response (**required**)

17.3 opdwhere

Where did you see the Label(**opdwho (17.1)** , Q(**opdwho (17.1)** ,0)) ?

Expects a single option response (**required**)

- Your own home [1]
- Local health centre [2]
- Private office [3]
- Hospital outpatient [4]

17.4 opdwhy

Why did you see the Label(**opdwho (17.1)** , Q(**opdwho (17.1)** ,0)) ?

Expects a single option response (**required**)

- Infectious disease (e.g. malaria) [1]
- Maternal / perinatal condition [2]
- Acute condition (e.g. flu, cough) [3]
- Injury [4]
- Sleep problems [5]
- Depression or anxiety [6]
- Alcohol problems [7]
- Other mental health problems [8]
- Other chronic disease (e.g. heart, diabetes) [9]
- Other [77]
- Don't Know [888]

Prerequisites
Skip when *opdwhy (17.4)* Not Equal 'Other [77]'

17.5 opdwhyo

Please specify why you saw the Label(**opdwho (17.1)** , Q(**opdwho (17.1)** ,0)) :

Expects a single line text response (**required**)

17.6 opdfeat

What were the main features of the visit to the Label(**opdwho (17.1)** , Q(**opdwho (17.1)** ,0)) ? [Select up to THREE features]

Expects multiple selected options (**required**)

- Assessment and/or diagnosis [1]
- Drug prescription (drug for condition listed in the previous answer) [2]
- Drug prescription (for other condition) [3]
- Psychosocial support / care [4]
- Follow-up visit [5]
- Referral (to other provider) [6]
- Other [77]
- Don't Know [888]

17.7 reprocessnumberoffeatures

Operator

This field is not displayed on the handset, Operator: Reprocess(**numberoffeatures (1.17)**)

17.8 setnumberoffeatures

Operator

This field is not displayed on the handset, Operator: Set(**numberoffeatures (1.17)** , Count(**opdfeat (17.6)**))

Prerequisites
Skip when *numberoffeatures (1.17)* Less Than '4'

17.9 toomanyfeatures

[You have identified __{377484}__ features of the clinical visit. Enter only the THREE main features of the clinical visit.]

Expects a single option response (**required**)

- Go back [1]

Branches

If response **Equals 'Go back [1]'** then skip to *opdfeat (17.6)*

Prerequisites
Skip when *opdfeat (17.6)* Excludes 'Other [77]'

17.10 opdfeato

Please specify the other feature of the visit to the Label(**opdwho (17.1)** , Q(**opdwho (17.1)** ,0)) :

Expects a single line text response (**required**)

Prerequisites
Skip when *opdfeat (17.6)* Excludes 'Referral (to other provider) [6]'

17.11 opdreferral

To whom did you get a referral?

Expects multiple selected options (**required**)

- Traditional healer/spiritualist/herbalist [1]
- Community health worker [2]
- Nurse/midwife [3]
- Pharmacist [4]
- General doctor [5]
- Specialist doctor [6]
- Psychiatrist [7]
- Counsellor [8]
- Psychiatric nurse [9]
- Social worker [10]
- Religious or spiritual advisor [11]
- Group counselling [12]
- Other [77]
- Don't know [888]

17.12 opdnarrative

Brief narrative about the content of the treatment, including the number of previous and future visits.

Expects a single line text response (**required**)

17.13 opdsatis

How satisfied are you with the treatment you received from the Label(**opdwho (17.1)** , Q(**opdwho (17.1)** ,0)) ?

Expects a single option response (**required**)

- Very satisfied [1]
- Satisfied [2]
- Neither satisfied or dissatisfied [3]
- Dissatisfied [4]
- Very dissatisfied [5]
- Don't know [888]

17.14 opdtravel

How long did it take you to travel to the Label(**opdwho (17.1)** , Q(**opdwho (17.1)** ,0)) ? [Enter number of MINUTES, one direction]

Expects a numeric response (**required**)

17.15 opdwait

How long did you wait for your consultation with the Label(**opdwho (17.1)** , Q(**opdwho (17.1)** ,0)) ? [Enter number of MINUTES]

Expects a numeric response (**required**)

17.16 opdlong

How long was the consultation with the Label(**opdwho (17.1)** , Q(**opdwho (17.1)** ,0)) (excluding waiting time)? [Enter consultation time in MINUTES]

Expects a numeric response (**required**)

17.17 opdfees

How much did you, your family or friends have to pay for the consultation with the Label(**opdwho (17.1)** , Q(**opdwho (17.1)** ,0)) ? [Enter fees in __{LABEL(375774,__{374020}__)}__ units]

Expects a decimal response (**required**)

17.18 opdfare

How much did you, your family or friends have to pay for travel? [Enter travel cost in __{LABEL(375774,__{374020}__)}__ units]

Expects a decimal response (**required**)

Section Prerequisites
Skip when *participant (3.3)* Equals'Caregiver [1]'

Section 18. PACIC

18.1 pacic instructions 1

Staying healthy can be difficult when you have Label(**cohort (3.2)** , Q(**cohort (3.2)** ,0)) . I would like to learn about the type of help you get from health workers for Label(**cohort (3.2)** , Q(**cohort (3.2)** ,0)) . This might include a health extension worker in a health post, a nurse or health officer in a health centre or a doctor in the hospital. Label(**cohort (3.2)** , Q(**cohort (3.2)** ,0))

18.2 pacicany

In the past 3 months, have you received any health care for problems with Label(**cohort (3.2)** , Q(**cohort (3.2)** ,0)) ?

Expects a single option response (**required**)

- No [0]
- Yes [1]

Branches

If response **Equals 'No [0]'** then skip to ***endofpacic (18.15)***

Prerequisites
Skip when ***country (3.1)*** **Equals 'South Africa - Dr KK [4]'**

18.3 pacic instructions 2

Over the past 3 months (since __{377429}__), when you received care for Label(**cohort (3.2)** , Q(**cohort (3.2)** ,0)) how often were you...

Prerequisites
Skip when ***country (3.1)*** **Not Equal 'South Africa - Dr KK [4]'**

18.4 pacic instructions 2_SA

Over the past 3 months (since __{377429}__), when you received care for Label(**cohort (3.2)** , Q(**cohort (3.2)** ,0)) how often were you... [Show FLASH CARD 6]

18.5 pacic_org

How often were you... Satisfied that your care was well organized, for example, that there was good coordination between different providers involved with your care?

Expects a single option response (**required**)

- None of the time [1]
- A little of the time [2]
- Some of the time [3]
- Most of the time [4]
- Always [5]

18.6 pacic_care

How often were you... Encouraged by providers to take more care of your condition, for example, shown how any actions that you have taken to take care of yourself has improved your condition?

Expects a single option response (**required**)

- None of the time [1]
- A little of the time [2]
- Some of the time [3]
- Most of the time [4]
- Always [5]

18.7 pacic_setup

How often were you... Helped to set specific goals to improve your condition, e.g., getting active, acting on a problem, joining a support group?

Expects a single option response (**required**)

- None of the time [1]
- A little of the time [2]
- Some of the time [3]
- Most of the time [4]
- Always [5]

18.8 pacic_values

How often were you... Asked about your understanding of Label(**cohort (3.2)** , Q(**cohort (3.2)** ,0)) and whether the treatment/interventions recommended were in alignment with your values, beliefs and traditions?

Expects a single option response (**required**)

- None of the time [1]
- A little of the time [2]
- Some of the time [3]
- Most of the time [4]
- Always [5]

18.9 pacic_skills

How often were you... Provided skills to help you to deal with your condition even when under great stress or hard times?

Expects a single option response (**required**)

- None of the time [1]
- A little of the time [2]
- Some of the time [3]
- Most of the time [4]
- Always [5]

18.10 pacic_community

How often were you... Encouraged to attend programs in the community that could help you?

Expects a single option response (**required**)

- None of the time [1]
- A little of the time [2]
- Some of the time [3]
- Most of the time [4]
- Always [5]

18.11 pacic_refer

How often were you... Referred to a counsellor or equivalent who could help you with Label(**cohort (3.2)** , Q(**cohort (3.2)** ,0)) ?

Expects a single option response (**required**)

- None of the time [1]
- A little of the time [2]
- Some of the time [3]
- Most of the time [4]
- Always [5]

18.12 pacic_going

How often were you... Asked how your visits with other providers were going?

Expects a single option response (**required**)

- None of the time [1]
- A little of the time [2]
- Some of the time [3]
- Most of the time [4]
- Always [5]

18.13 pacic_info

How often were you... Given information and education about Label(**cohort (3.2)** , Q(**cohort (3.2)** ,0)) ?

Expects a single option response (**required**)

- None of the time [1]
- A little of the time [2]
- Some of the time [3]
- Most of the time [4]
- Always [5]

18.14 pacic_follow

How often were you... Given follow up appointments for treatment of Label(**cohort (3.2)** , Q(**cohort (3.2)** ,0)) ?

Expects a single option response (**required**)

- None of the time [1]
- A little of the time [2]
- Some of the time [3]
- Most of the time [4]
- Always [5]

18.15 endofpacic

[End of PACIC section]

Section Prerequisites
Skip when *Participant-proxy (1.4)* Equals'3'

Section 19. Medications

Prerequisites
Skip when *country (3.1)* Not Equal 'Uganda - Kamuli [5]'

19.1 medi

[These question must be answered by the caregiver on behalf of the patient - the following questions refer to the patient's medication.]

19.2 rx_instructions

[Instruction for country teams: List all known psychotropic medications being used in any PRIME country and list them for use in Question 2. Non-psychotropic will fall under Other (specify)=77]

19.3 rxmeds

In the past THREE months (since __{377429}__), how many new or continuing medications have you been prescribed for ANY health condition?

Expects a numeric response (**required**)

Repeat this section for value of ***rxmeds (19.3)***

Section Prerequisites
Skip when ***Participant-proxy (1.4)*** **Equals'3'**

Section 20. Medication repeat

20.1 rxname

What is the name of the __{LABEL(375641,REPEAT IDX medication you were prescribed? If you do not remember what medication you have been prescribed you can show me the pills or prescriptions.

Expects a single option response (**required**)

- Amytryptyline [15]
- Carbemazepine [2]
- Diazapam [4]
- Fluoxetine [1]
- Haloperidol [10]
- Imipramine [17]
- Lamotrigine [8]
- Orphenadrine [11]
- Phenobarbitol [14]
- Phenytoine [9]
- Promethazine [18]
- Resperidone [5]
- Sodium valproate [7]
- Thiamine [3]
- Trihexiphenyidil [6]
- Other medication [77]

Prerequisites
Skip when ***rxname (20.1)*** **Not Equal 'Other medication [77]'**

20.2 rxnameo

What is the name of the other medication?

Expects a single line text response (**required**)

20.3 rxdays

For how many days was Label(**rxname (20.1)** , Q(**rxname (20.1)** ,0)) most recently prescribed? [Don't know=88]

Expects a numeric response (**required**)

20.4 rxdose

What was the daily dosage for Label(**rxname (20.1)** , Q(**rxname (20.1)** ,0)) ? [Enter the milligrams / entire day. Note: 1g = 1000 mg.] [Don't know = 88]

Expects a numeric response (**required**)

20.5 rxwho

Who prescribed the Label(**rxname (20.1)** , Q(**rxname (20.1)** ,0)) for you?

Expects a single option response (**required**)

- Traditional healer [1]
- Religious/spiritual advisor [2]
- Community health worker [3]
- Nurse/midwife [4]
- Pharmacist [5]
- General doctor [6]
- Specialist doctor (non-psychiatrist) [7]
- Psychiatrist [8]
- Other mental health worker [9]
- Other [77]

Prerequisites
Skip when ***rxwho (20.5)*** **Not Equal 'Other [77]'**

20.6 rxwhoo

Specify who prescribed the Label(**rxname (20.1)** , Q(**rxname (20.1)** ,0))

Expects a single line text response (**required**)

Prerequisites
Skip when ***rxname (20.1)*** **Not Equal 'Other medication [77]'**

20.7 notpsychotropic-skiptonext

[This drug is not a psychotropic. Skip ahead.]

Expects a single option response (optional)

- Next [1]

Branches

If response **Equals 'Next [1]'** then skip to ***endofmedications (20.15)***

20.8 rxforget

Do you forget to take Label(**rxname (20.1)** , Q(**rxname (20.1)** ,0)) ?

Expects a single option response (**required**)

- No [0]
- Yes [1]

20.9 rxcareless

Are you careless at times about taking the Label(**rxname (20.1)** , Q(**rxname (20.1)** ,0)) ?

Expects a single option response (**required**)

- No [0]
- Yes [1]

20.10 rxbetter

When you feel better do you sometimes stop taking Label(**rxname (20.1)** , Q(**rxname (20.1)** ,0)) ?

Expects a single option response (**required**)

- No [0]
- Yes [1]

20.11 rxworse

Sometimes if you feel worse when you take Label(**rxname (20.1)** , Q(**rxname (20.1)** ,0)) , do you stop taking it?

Expects a single option response (**required**)

- No [0]
- Yes [1]

20.12 rxmonth

In the past ONE MONTH (since __{377317}__), have you been taking the Label(**rxname (20.1)** , Q(**rxname (20.1)** ,0)) as prescribed by the Label(**rxwho (20.5)** , Q(**rxwho (20.5)** ,0)) ?

Expects a single option response (**required**)

- All the time [1]
- Most of the time (>3 of the last 4 weeks) [2]
- Sometimes (at least 2-3/4 weeks) [3]
- Took the medicine occasionally (<2 of the last 4 weeks) [4]
- Did not take any medicine at all [5]

20.13 rxadvice

Did the Label(**rxwho (20.5)** , Q(**rxwho (20.5)** ,0)) give you advice and support with taking Label(**rxname (20.1)** , Q(**rxname (20.1)** ,0)) ?

Expects a single option response (**required**)

- No [0]
- Yes [1]
- Don't know/Can't remember [88]

20.14 rxsfx

Did the Label(**rxwho (20.5)** , Q(**rxwho (20.5)** ,0)) talk to you about potential side effects of Label(**rxname (20.1)** , Q(**rxname (20.1)** ,0)) and what to do if you experience any of those side effects?

Expects a single option response (**required**)

- No [0]
- Yes [1]
- Don't know/Can't remember [88]

20.15 endofmedications

[End of this medication]

Section Prerequisites
Skip when *participant (3.3)* Equals'Patient [2]' OR
Skip when *cohort (3.2)* Equals'maternal depression [2]'

Section 21. Covert Medication

21.1 takingmeds

Overall, in the past 1 month (since __{377317}__), do you think that Q**ptname (4.17)** has been taking the medicines as prescribed by the health worker?

Expects a single option response (**required**)

- All the time [1]
- Most of the time (>3 of the last 4 weeks) [2]
- Sometimes (at least 2-3/4 weeks) [3]
- Took the medicine occasionally (<2 of the last 8 weeks) [4]
- Did not take any medicine at all [5]

21.2 covertmed

Sometimes when a relative is unwell or is unwilling to take their medication, family members give the medication without the relatives knowledge. For example by putting the medication into the relatives food. Have you or anyone in your family ever given Q**ptname (4.17)** medication without their knowledge?

Expects a single option response (**required**)

- No [0]
- Yes [1]

Section Prerequisites
Skip when ***participant (3.3)*** **Equals'Caregiver [1]'** OR
Skip when ***country (3.1)*** **Equals'South Africa - Dr KK [4]'**

Section 22. CGI - Community Group Interventions

22.1 groupinterventions

For treatment of Label(**cohort (3.2)** , Q(**cohort (3.2)** ,0)) in the past THREE months, have you been involved in any of the following: Tell me yes or no as I list the options.

Expects multiple selected options (**required**)

- [ ] THP Thinking Healthy Programme [1]
- [ ] CAP Counseling for Alcohol Problem [2]
- [ ] FM Family Intervention [3]
- [ ] HAP Healthy Activitiy Program [5]
- [ ] Basic Psychosocial Counseling [6]
- [ ] Alcoholics anonymous [7]
- [ ] Group psychosocial counselling [8]
- [ ] Peer support group [13]
- [ ] Not involved with any of the above [0]

Section Prerequisites
Skip when ***participant (3.3)*** **Equals'Caregiver [1]'** OR
Skip when ***country (3.1)*** **Not Equal'South Africa - Dr KK [4]'**

Section 23. CGI - SA

23.1 groupinterventions_sa

For treatment of Label(**cohort (3.2)** , Q(**cohort (3.2)** ,0)) in the past THREE months, have you been involved in any of the following: Tell me yes or no as I list the options.

Expects multiple selected options (**required**)

- ☐ Alcoholics anonymous [7]
- ☐ Individual depression counselling [8]
- ☐ Advice for alcohol problems [9]
- ☐ Group depression counseling [10]
- ☐ Psychosocial rehabilitation group treatment [11]
- ☐ Not involved with any of the above [0]

Section Prerequisites
Skip when ***groupinterventions_sa (23.1)*** **Excludes'Psychosocial rehabilitation group treatment [11]'**

Section 24. CGI-PSR

24.1 psr_seen

How many PSR group sessions have you attended in the past 3 months?

Expects a numeric response (**required**)

24.2 psr_mins

How long were the PSR group sessions on average? (minutes)

Expects a numeric response (**required**)

24.3 psr_cont

Are you still involved with the PSR treatment?

Expects a single option response (**required**)

- No [0]
- Yes [1]

24.4 psr_who

Who facilitated the PSR group sessions?

Expects a single option response (**required**)

- Prescriber [1]
- Non-prescriber [2]
- Community counselor [3]

24.5 psr_help

How much did the PSR group sessions help you?

Expects a single option response (**required**)

- A lot [1]
- Some [2]
- A little [3]
- Not at all [4]
- Don't know [88]

Section Prerequisites
Skip when ***groupinterventions (22.1)*** **Excludes'Basic Psychosocial Counseling [6]'**

Section 25. CGI-Basic

25.1 basic_seen

How many BASIC meetings have you attended in the past 3 months?

Expects a numeric response (**required**)

25.2 basic_mins

How long were the BASIC meetings on average? (minutes)

Expects a numeric response (**required**)

25.3 basic_cont

Are you still involved with the BASIC?

Expects a single option response (**required**)

- No [0]
- Yes [1]

25.4 basic_who

Who facilitated the sessions for the BASIC?

Expects a single option response (**required**)

- Prescriber [1]
- Non-prescriber [2]
- Community counselor [3]

25.5 basic_help

How much did the BASIC Programme help you?

Expects a single option response (**required**)

- A lot [1]
- Some [2]
- A little [3]
- Not at all [4]
- Don't know [88]

Section Prerequisites
Skip when ***groupinterventions (22.1)*** **Excludes'HAP Healthy Activitiy Program [5]'**

Section 26. CGI-HAP

26.1 hap_seen

How many HAP meetings have you attended in the past 3 months?

Expects a numeric response (**required**)

26.2 hap_mins

How long were the HAP meetings on average? (minutes)

Expects a numeric response (**required**)

26.3 hap_cont

Are you still involved with the HAP?

Expects a single option response (**required**)

- No [0]
- Yes [1]

26.4 hap_who

Who facilitated the sessions for the HAP?

Expects a single option response (**required**)

- Prescriber [1]
- Non-prescriber [2]
- Community counselor [3]

26.5 hap_help

How much did the HAP help you?

Expects a single option response (**required**)

- A lot [1]
- Some [2]
- A little [3]
- Not at all [4]
- Don't know [88]

Section Prerequisites
Skip when ***groupinterventions (22.1)*** **Excludes'CAP Counseling for Alcohol Problem [2]'**

Section 27. CGI-CAP

27.1 cap_seen

How many CAP meetings have you attended in the past 3 months?

Expects a numeric response (**required**)

27.2 cap_mins

How long were the CAP meetings on average? (minutes)

Expects a numeric response (**required**)

27.3 cap_cont

Are you still involved with the CAP?

Expects a single option response (**required**)

- No [0]
- Yes [1]

27.4 cap_who

Who facilitated the sessions for the CAP?

Expects a single option response (**required**)

- Prescriber [1]
- Non-prescriber [2]
- Community counselor [3]

27.5 cap_help

How much did the CAP help you?

Expects a single option response (**required**)

- A lot [1]
- Some [2]
- A little [3]
- Not at all [4]
- Don't know [88]

Section Prerequisites
Skip when ***groupinterventions (22.1)*** **Excludes'THP Thinking Healthy Programme [1]'**

Section 28. CGI-THP

28.1 thp_seen

How many meetings have you attended in the past 3 months?

Expects a numeric response (**required**)

28.2 thp_mins

How long were the meetings on average? (minutes)

Expects a numeric response (**required**)

28.3 thp_cont

Are you still involved with the Thinking Healthy Programme?

Expects a single option response (**required**)

- No [0]
- Yes [1]

28.4 thp_who

Who facilitated the sessions for the Thinking Healthy Programme?

Expects a single option response (**required**)

- Prescriber [1]
- Non-prescriber [2]
- Community counselor [3]

28.5 thp_help

How much did the Thinking Healthy Programme help you?

Expects a single option response (**required**)

- A lot [1]
- Some [2]
- A little [3]
- Not at all [4]
- Don't know [88]

Section Prerequisites
Skip when ***groupinterventions (22.1)*** **Excludes'Group psychosocial counselling [8]'**

Section 29. CGI-GPC

29.1 gpc_seen

How many group psycho-social counselling sessions have you attended in the past 3 months?

Expects a numeric response (**required**)

29.2 gpc_mins

How long were the group psychosocial counselling sessions on average?

Expects a numeric response (**required**)

29.3 gpc_cont

Are you still involved with the group psychosocial counselling?

Expects a single option response (**required**)

- No [0]
- Yes [1]

29.4 gpc_who

Who facilitated the sessions for the group psychosocial counselling?

Expects a single option response (**required**)

- Prescriber [1]
- Non-prescriber [2]
- Community counselor [3]

29.5 gpc_help

How much did the group psychosocial counselling help you?

Expects a single option response (**required**)

- A lot [1]
- Some [2]
- A little [3]
- Not at all [4]
- Don't know [88]

Section Prerequisites
Skip when ***groupinterventions (22.1)*** **Excludes'Peer support group [13]'**

Section 30. CGI-PSG

30.1 psg_seen

How many peer support group meetings have you attended in the past 3 months?

Expects a numeric response (**required**)

30.2 psg_mins

How long were the peer support group meetings on average?

Expects a numeric response (**required**)

30.3 psg_cont

Are you still involved with the peer support group ?

Expects a single option response (**required**)

- No [0]
- Yes [1]

30.4 psg_who

Who facilitated the peer support group meetings?

Expects a single option response (**required**)

- Prescriber [1]
- Non-prescriber [2]
- Community counselor [3]

30.5 psg_help

How much did the peer support group help you?

Expects a single option response (**required**)

- A lot [1]
- Some [2]
- A little [3]
- Not at all [4]
- Don't know [88]

Section Prerequisites
Skip when ***groupinterventions_sa (23.1)*** **Excludes'Group depression counseling [10]'**

Section 31. CGI-Group

31.1 group_seen

How many depression group counseling meetings have you attended in the past 3 months?

Expects a numeric response (**required**)

31.2 group_mins

How long were thedepression group counseling meetings on average? (minutes)

Expects a numeric response (**required**)

31.3 group_cont

Are you still involved with depression group counseling?

Expects a single option response (**required**)

- No [0]
- Yes [1]

31.4 group_who

Who facilitated the sessions for the group counselling?

Expects a single option response (**required**)

- Prescriber [1]
- Non-prescriber [2]
- Community counselor [3]

31.5 group_help

How much did depression group counseling help you?

Expects a single option response (**required**)

- A lot [1]
- Some [2]
- A little [3]
- Not at all [4]
- Don't know [88]

Section Prerequisites
Skip when ***groupinterventions_sa (23.1)*** **Excludes'Individual depression counselling [8]'**

Section 32. CGI-IDC

32.1 idc_seen

How many individual depression counseling meetings have you attended in the past 3 months?

Expects a numeric response (**required**)

32.2 idc_mins

How long were the individual depression counseling meetings on average? (minutes)

Expects a numeric response (**required**)

32.3 idc_cont

Are you still involved with individual depression counseling?

Expects a single option response (**required**)

- No [0]
- Yes [1]

32.4 idc_who

Who facilitated the sessions for the individual depression counseling?

Expects a single option response (**required**)

- Prescriber [1]
- Non-prescriber [2]
- Community counselor [3]

32.5 idc_help

How much did individual depression counseling help you?

Expects a single option response (**required**)

- A lot [1]
- Some [2]
- A little [3]
- Not at all [4]
- Don't know [88]

Section Prerequisites
Skip when ***groupinterventions_sa (23.1)*** **Excludes'Advice for alcohol problems [9]'**

Section 33. CGI-Alcohol advice

33.1 advice_seen

How many XXX meetings have you attended in the past 3 months?

Expects a numeric response (**required**)

Constraints

Response must be ***Greater Than or Equal'1'***

33.2 advice_mins

How long were the XXX meetings on average? (minutes)

Expects a numeric response (**required**)

33.3 advice_cont

Are you still involved with XXX?

Expects a single option response (**required**)

- No [0]
- Yes [1]

33.4 advice_who

Who facilitated the sessions for the XXX counselling?

Expects a single option response (**required**)

- Prescriber [1]
- Non-prescriber [2]
- Community counselor [3]

33.5 advice_help

How much did XXX help you?

Expects a single option response (**required**)

- A lot [1]
- Some [2]
- A little [3]
- Not at all [4]
- Don't know [88]

Section Prerequisites
Skip when ***groupinterventions (22.1)*** **Excludes'Alcoholics anonymous [7]'** AND
Skip when ***groupinterventions_sa (23.1)*** **Excludes'Alcoholics anonymous [7]'**

Section 34. CGI-AA

34.1 aa_seen

How many AA meetings have you attended in the past 3 months?

Expects a numeric response (**required**)

34.2 aa_mins

How long were the AA meetings on average? (minutes)

Expects a numeric response (**required**)

34.3 aa_cont

Are you still involved with AA?

Expects a single option response (**required**)

- No [0]
- Yes [1]

34.4 aa_who

Who facilitated the sessions for AA?

Expects a single option response (**required**)

- Prescriber [1]
- Non-prescriber [2]
- Community counselor [3]

34.5 aa_help

How much did AA help you?

Expects a single option response (**required**)

- A lot [1]
- Some [2]
- A little [3]
- Not at all [4]
- Don't know [88]

Section Prerequisites
Skip when *visit (3.7)* **Equals'Baseline [1]'** OR
Skip when *participant (3.3)* **Equals'Caregiver [1]'**

Section 35. MH treatment received

35.1 mhtreat_instruct1

In this section, I'm going to ask you a few more questions about the care you have received over the past THREE months, that is since __{377429}__, for your problems with Label(**cohort (3.2)** , Q(**cohort (3.2)**)) specifically.

Prerequisites
Skip when *visit (3.7)* **Not Equal 'Midline [2]'**

35.2 aware_ml

Are you aware that you were identified with problems of Label(**cohort (3.2)** , Q(**cohort (3.2)**)) three months ago?

Expects a single option response (**required**)

- No [0]
- Yes [1]

Prerequisites
Skip when *visit (3.7)* **Not Equal 'Endline [3]'**

35.3 aware_el

Are you aware that you were identified with problems of Label(**cohort (3.2)** , Q(**cohort (3.2)**)) one year ago?

Expects a single option response (**required**)

- No [0]
- Yes [1]

35.4 hcenter

Did you visit the health centre for this problem?

Expects a single option response (**required**)

- No [0]
- Yes [1]

Prerequisites
Skip when *hcenter (35.4)* **Equals 'No [0]'**

35.5 hcentrecount

How many times did you visit the health centre for this problem? [Ask for an estimate]

Expects a numeric response (**required**)

35.6 treattype

What treatment did you receive for this problem?

Expects multiple selected options (**required**)

- Medicine [1]
- Psychosocial support from non-prescribers/nurses [2]
- Psychosocial support/counselling from counselors/auxiliary social workers [3]
- Home-based care from FCHVs/HBCWs [4]
- Home-based care from counselors [5]
- Psychological care from a psychologist [6]
- None [0]

Prerequisites
Skip when *treattype (35.6)* **Excludes 'Psychosocial support/counselling from counselors/auxiliary social workers [3]'** OR
Skip when *country (3.1)* **Not Equal 'South Africa - Dr KK [4]'**

35.7 psysocsess

How many psychosocial sessions with the counsellor/auxiliary social worker did you attend?

Expects a numeric response (**required**)

Prerequisites
Skip when *treattype (35.6)* **Includes 'None [0]'**

35.8 treattypeo

What other treatments did you receive for this problem?

Expects multiple selected options (**required**)

- Traditional healer [1]
- Spiritual/religious leader [2]
- Other communtiy health worker [3]
- Support group [4]
- Other [77]
- None [0]

Prerequisites
Skip when *treattypeo (35.8)* **Excludes 'Other [77]'** OR
Skip when *treattype (35.6)* **Includes 'None [0]'**

35.9 treattypeoo

[Specify what other treatment was received]

Expects a single line text response (**required**)

Prerequisites
Skip when *treattype (35.6)* **Excludes 'Medicine [1]'**

35.10 treatmed

What medicine did you take?

Expects multiple selected options (**required**)

- Fluoxetine [1]
- Carbemazepine [2]
- Thiamine [3]
- Diazepam [4]
- Resperidone [5]
- Trihexiphynidil [6]
- Sodium valproate [7]
- Lamotrigine [8]
- Phenytoine [9]
- Haloperidol [10]
- Orphenadrine [11]
- Flupenthixol [12]
- Amytryptyline [15]
- Imipramine [17]
- Olanzapine [19]
- Alprazolam [20]
- Phenobarbitone [21]
- Chlorpromazine [22]
- Lorazepam [23]
- Chlordiazepoxide [24]
- Escitalopram [25]
- Sertaline [26]
- Staycam plus [27]
- Quetiapine [28]
- Other medication [77]

Prerequisites
Skip when ***treatmed (35.10)*** **Excludes 'Other medication [77]'**

35.11 treatmedo

[Specify which other medication]

Expects a single line text response (**required**)

Section Prerequisites
Skip when ***cohort (3.2)*** **Not Equal'maternal depression [2]'** OR
Skip when ***country (3.1)*** **Not Equal'Uganda - Kamuli [5]'**

Section 36. AAS

36.1 aas_ever

Have you ever been emotionally or physically abused by your partner or someone important to you?

Expects a single option response (**required**)

- No [0]
- Yes [1]

36.2 aas_physhurt

Within the last year, have you ever been hit, slapped, kicked, or otherwise physically hurt by someone?

Expects a single option response (**required**)

- No [0]
- Yes [1]

Branches
If response **Equals 'No [0]'** then skip to ***aas_preghurt (36.6)***

36.3 aas_hurtwho

Who physically hurt you?

Expects multiple selected options (**required**)

- Husband [1]
- Ex-husband [2]
- Boyfriend [3]
- Stranger [4]
- Other [77]

Prerequisites
Skip when ***aas_hurtwho (36.3)*** **Excludes 'Other [77]'**

36.4 aas_hurtwhoo

[Please specify other]

Expects a single line text response (**required**)

36.5 aas_hurttimes

How many times were you physically hurt in the past year?

Expects a numeric response (**required**)

36.6 aas_preghurt

Since you've been pregnant, have you been slapped, kicked, or otherwise physically hurt by someone?

Expects a single option response (**required**)

- No [0]
- Yes [1]

Branches
If response **Equals 'No [0]'** then skip to ***aas_sexforce (36.12)***

36.7 aas_preghurtwho

Who physically hurt you?

Expects multiple selected options (**required**)

- Husband [1]
- Ex-husband [2]
- Boyfriend3 [Boyfriend3]
- Stranger [4]
- Other [77]

Prerequisites
Skip when *aas_preghurtwho (36.7)* **Excludes 'Other [77]'**

36.8 aas_preghurtwhoo

[Please specify other]

Expects a single line text response (**required**)

36.9 aas_preghurttimes

How many times were you physically hurt during this pregnancy?

Expects a numeric response (**required**)

36.10 aas_where

Indicate the area(s) of injury

Expects a long text response (**required**)

36.11 aas_mostsevere

Score the most severe incident during your pregnancy to the following scale:

Expects a single option response (**required**)

- Threats of abuse including use of weapon [1]
- Slapping, pushing; no injuries and/or lasting pain [2]
- Punching, kicking, bruises, cuts and/or continuing pain [3]
- Beating up, severe contusions, burns, broken bones [4]
- Head injury, internal injury, permanent injury [5]
- Use of weapon; wound from weapon [6]

36.12 aas_sexforce

Within the last year, has anyone forced you to have sexual activities?

Expects a single option response (**required**)

- No [0]
- Yes [1]

Branches
If response **Equals 'No [0]'** then skip to ***aas_afraid (36.16)***

36.13 aas_forcesexwho

Who forced you to have sexual activities?

Expects multiple selected options (**required**)

- Husband [1]
- Ex-husband [2]
- Boyfriend [3]
- Stranger [4]
- Other [77]

Prerequisites
Skip when *aas_forcesexwho (36.13)* **Excludes 'Other [77]'**

36.14 aas_forcesexwhoo

[Please specify]

Expects a single line text response (**required**)

36.15 aas_forcesextimes

How many times were you forced to have sexual activities in the past year?

Expects a numeric response (**required**)

36.16 aas_afraid

Are you afraid of your partner or anyone listed above?

Expects a single option response (**required**)

- No [0]
- Yes [1]

Section 37. Economic Activity

37.1 occ

What is your main occupational status?

Expects a single option response (**required**)

- Home worker (e.g. housewife) [1]
- Unskilled labour (farmhand / domestic worker / subsistence farmer) [2]
- Skilled labour (builder) [3]
- Services-sales (shop worker) [4]
- Clerical worker (secretary) [5]
- Professional (nurse, lawyer, doctor) [6]
- Student [7]
- Retired-pensioned [8]
- Other [77]

Prerequisites
Skip when ***occ (37.1)*** **Not Equal 'Other [77]'**

37.2 occo

Specify the occupational status:

Expects a single line text response (**required**)

37.3 empnow

What is your CURRENT employment status?

Expects a single option response (**required**)

- Self-employed (including subsistence farmer) [1]
- Full- or part-time employment by someone else (wage or salaried) [2]
- Voluntarily employed (unpaid) [3]
- Not employed (including housewife) [4]

Branches

If response **Equals 'Voluntarily employed (unpaid) [3]'** then skip to ***econworkhrs (37.6)***

If response **Equals 'Not employed (including housewife) [4]'** then skip to ***empever (37.8)***

Prerequisites
Skip when ***empever (37.8)*** **Equals 'No [0]'**

37.4 econearn

How much do you usually earn (before taxes and other deductions)? [Enter salary in __{LABEL(375774, __{374020}__)}__ units]

Expects a numeric response (**required**)

37.5 econpayment

[Enter frequency of earnings, for the previous answer]

Expects a single option response (**required**)

- per day [1]
- per week [2]
- per month [3]
- per year [4]

37.6 econworkhrs

About how many hours did you work in the past 4 weeks (28 days)?

Expects a numeric response (**required**)

37.7 gotowhodas

Operator

This field is not displayed on the handset, Operator: GoTo(**ECON-WHODAS (37.13)**)

37.8 empever

Have you EVER worked for income / had employment?

Expects a single option response (**required**)

- No [0]
- Yes [1]

Branches

If response **Equals 'No [0]'** then skip to ***econreturn (37.12)***

Prerequisites
Skip when ***empever (37.8)*** **Equals 'Yes [1]'**

37.9 econunempy

How long ago did you LAST work for income / have employment? [Enter number of YEARS] [Enter 0 if unemployment is <1 year]

Expects a numeric response (**required**)

Prerequisites
Skip when ***empever (37.8)*** **Equals 'Yes [1]'** OR
Skip when ***econunempy (37.9)*** **Greater Than '0'**

37.10 econunempm

How long ago did you LAST work for income / have employment? [Enter 1 to 11 MONTHS] [Enter 0 if unemployment is <1 month]

Expects a numeric response (**required**)

Constraints

Response must be ***Less Than or Equal'11'***

Prerequisites
Skip when ***empever (37.8)*** **Equals 'Yes [1]'** OR
Skip when ***econunempm (37.10)*** **Greater Than '0'**

37.11 econunempd

How long ago did you LAST work for income / have employment? [Enter 1 to 30 DAYS]

Expects a numeric response (**required**)

Constraints

Response must be ***between'1' and '30'***

Prerequisites
Skip when ***empever (37.8)*** **Equals 'Yes [1]'**

37.12 econreturn

Are you looking for work or intending to return to work?

Expects a single option response (**required**)

- No [0]
- Yes [1]

37.13 ECON-WHODAS

For your HOUSEHOLD tasks... In the past 30 days how much difficulty did you have in...

37.14 econhhwell

How much difficulty did you have in... Doing your most important household tasks well?

Expects a single option response (**required**)

- None [1]
- Mild [2]
- Moderate [3]
- Severe [4]
- Extreme or cannot do [5]

37.15 econdone

How much difficulty did you have in... Getting all the household work done that you needed to do?

Expects a single option response (**required**)

- None [1]
- Mild [2]
- Moderate [3]
- Severe [4]
- Extreme or cannot do [5]

37.16 eapquickly

How much difficulty did you have in... Getting your household work done as quickly as needed?

Expects a single option response (**required**)

- None [1]
- Mild [2]
- Moderate [3]
- Severe [4]
- Extreme or cannot do [5]

Prerequisites
Skip when ***participant (3.3)*** **Equals 'Caregiver [1]'**

37.17 eapmiss

In the past 30 days, on how many days did you reduce or completely miss household work because of Label(**cohort (3.2)** , Q(**cohort (3.2)** ,0)) ? [Enter 0 to 30 DAYS]

Expects a numeric response (**required**)

Constraints

Response must be ***between'0' and '30'***

Prerequisites
Skip when ***participant (3.3)*** **Equals 'Caregiver [1]'**

37.18 eap EA Instructions 2

For your MOST IMPORTANT work/tasks.... Because of Label(**cohort (3.2)** , Q(**cohort (3.2)** ,0)) , in the past 30 days, how much difficulty did you have in:

Prerequisites
Skip when ***participant (3.3)*** **Equals 'Caregiver [1]'**

37.19 eaptaskswell

How much difficulty did you have in... Doing your most important work/tasks well?

Expects a single option response (**required**)

- None [1]
- Mild [2]
- Moderate [3]
- Severe [4]
- Extreme/cannot do [5]

Prerequisites
Skip when ***participant (3.3)*** **Equals 'Caregiver [1]'**

37.20 eaptasksdone

How much difficulty did you have in...Getting all the important work/tasks done that you need to do?

Expects a single option response (**required**)

- None [1]
- Mild [2]
- Moderate [3]
- Severe [4]
- Extreme/cannot do [5]

Prerequisites
Skip when ***participant (3.3)*** **Equals 'Caregiver [1]'**

37.21 eaptasksquick

How much difficulty did you have in... Getting your important work/tasks done as quickly as needed?

Expects a single option response (**required**)

- None [1]
- Mild [2]
- Moderate [3]
- Severe [4]
- Extreme/cannot do [5]

37.22 hhfood

Has anyone in your household, including yourself, been hungry in the last month due to lack of resources/food?

Expects a single option response (**required**)

- No [0]
- Yes [1]

Section Prerequisites
Skip when *participant (3.3)* **Equals'Caregiver [1]'**

Section 38. OSLO

38.1 oslo Instruction 1

The following 3 questions ask about how you experience your social relationships. The inquiry is about your immediate personal experience.

38.2 oslo Instruction 2

Please indicate the option that represents your experience. If you are not sure of your answer, try to do your best to give an answer.

38.3 oslocount

How many people are so close to you that you can count on them if you have serious personal problems?

Expects a single option response (**required**)

- None [1]
- 1 or 2 [2]
- 3-5 [3]
- More than 5 [4]

38.4 osloconcern

How much concern do people show in what you are doing?

Expects a single option response (**required**)

- A lot of concern and interest [5]
- Some concern and interest [4]
- Uncertain [3]
- Little concern and interest [2]
- No concern and interest [1]

38.5 oslohelp

How easy is it to get practical help from neighbors if you should need it?

Expects a single option response (**required**)

- Very easy [5]
- Easy [4]
- Possible [3]
- Difficult [2]
- Very difficult [1]

Section Prerequisites
Skip when ***country (3.1)*** **Not Equal'South Africa - Dr KK [4]'** OR
Skip when ***participant (3.3)*** **Equals'Caregiver [1]'** OR
Skip when ***visit (3.7)*** **Equals'Midline [2]'**

Section 39. Internalized Stigma

39.1 ISMII

You have mentioned that you frequently experience some problems with [your drinking/emotions] in the past year. I will ask you some questions about these problems. Let me know if you agree or disagree with the following statements.

39.2 ISMI01

I feel out of place in the world because of these problems

Expects a single option response (**required**)

- Strongly disagree [1]
- Disagree [2]
- Agree [3]
- Strongly agree [4]

39.3 ISMI05

I am embarrassed or ashamed of these problems

Expects a single option response (**required**)

- Strongly disagree [1]
- Disagree [2]
- Agree [3]
- Strongly agree [4]

39.4 ISMI16

I am disappointed in myself due to these problems

Expects a single option response (**required**)

- Strongly disagree [1]
- Disagree [2]
- Agree [3]
- Strongly agree [4]

39.5 ISMI17

These problems have spoiled my life

Expects a single option response (**required**)

- Strongly disagree [1]
- Disagree [2]
- Agree [3]
- Strongly agree [4]

39.6 ISMI19

Because of these problems, I need others to make most of my decisions for me

Expects a single option response (**required**)

- Strongly disagree [1]
- Disagree [2]
- Agree [3]
- Strongly agree [4]

39.7 ISMI23

I can't contribute anything to society because of these problems

Expects a single option response (**required**)

- Strongly disagree [1]
- Disagree [2]
- Agree [3]
- Strongly agree [4]

39.8 ISMI03

People discriminate against me due to these problems

Expects a single option response (**required**)

- Strongly disagree [1]
- Disagree [2]
- Agree [3]
- Strongly agree [4]

39.9 ISMI15

People often patronize me, or treat me like a child, just because of these problems

Expects a single option response (**required**)

- Strongly disagree [1]
- Disagree [2]
- Agree [3]
- Strongly agree [4]

39.10 ISMI22

People ignore me or take me less seriously just because of these problems

Expects a single option response (**required**)

- Strongly disagree [1]
- Disagree [2]
- Agree [3]
- Strongly agree [4]

39.11 ISMI25

Nobody would be interested in getting close to me because of these problems

Expects a single option response (**required**)

- Strongly disagree [1]
- Disagree [2]
- Agree [3]
- Strongly agree [4]

39.12 ISMI28

Others think that I can't achieve much in life because of these problems

Expects a single option response (**required**)

- Strongly disagree [1]
- Disagree [2]
- Agree [3]
- Strongly agree [4]

Section Prerequisites
Skip when ***participant (3.3)*** **Equals'Caregiver [1]'**

Section 40. Disclosure-participant

40.1 pdisclosure

In general, how happy would you feel talking to a friend or family member about your mental health, for example telling them you have a diagnosis of Label(**cohort (3.2)** , Q(**cohort (3.2)** ,0)) and how it affects you?

Expects a single option response (**required**)

- ☐ Very happy [1]
- ☐ Slightly happy [2]
- ☐ Slightly unhappy [3]
- ☐ Very unhappy [4]

Section Prerequisites
Skip when *cohort (3.2)* **Equals'depression [1]'** OR
Skip when *cohort (3.2)* **Equals'maternal depression [2]'** OR
Skip when *cohort (3.2)* **Equals'alcohol [3]'** OR
Skip when *participant (3.3)* **Equals'Caregiver [1]'** OR
Skip when *visit (3.7)* **Equals'Midline [2]'**

Section 41. DISC-12

41.1 DISC Instruction

In this section, I will ask you about times in the past YEAR when you may have been treated unfairly because of having Label(**cohort (3.2)** , Q(**cohort (3.2)** ,0)) .

41.2 disc_friend

Have you been treated unfairly in making or keeping friends?

Expects a single option response (**required**)

- Not at all [0]
- A little [1]
- Moderately [2]
- A lot [3]
- Not applicable [4]

41.3 disc_neighbour

Have you been treated unfairly by the people in your neighbourhood?

Expects a single option response (**required**)

- Not at all [0]
- A little [1]
- Moderately [2]
- A lot [3]
- Not applicable [4]

Prerequisites
Skip when *country (3.1)* **Equals 'Nepal - Chitwan [3]'**

41.4 disc_date

Have you been treated unfairly in dating or intimate relationships? [excluding treatment by spouse or co-habiting partner as covered by Q6]

Expects a single option response (**required**)

- Not at all [0]
- A little [1]
- Moderately [2]
- A lot [3]
- Not applicable [4]

41.5 disc_house

Have you been treated unfairly in housing? (including becoming homeless)

Expects a single option response (**required**)

- Not at all [0]
- A little [1]
- Moderately [2]
- A lot [3]
- Not applicable [4]

41.6 disc_educ

Have you been treated unfairly in your education? [Ask about school, college, university, on the job training, vocational courses]

Expects a single option response (**required**)

- Not at all [0]
- A little [1]
- Moderately [2]
- A lot [3]
- Not applicable [4]

41.7 disc_mar

Have you been treated unfairly in marriage or divorce? (including co-habiting or civil partnership.) [Ask about ability to find a partner or spouse, problems during the relationship, divorce settlements]

Expects a single option response (**required**)

- Not at all [0]
- A little [1]
- Moderately [2]
- A lot [3]
- Not applicable [4]

41.8 disc_fam

Have you been treated unfairly by your family? [Ask about family of origin parents, brothers, sisters and other relations as well as any children. Exclude treatment by spouse or co-habiting partner as covered by Q6]

Expects a single option response (**required**)

- Not at all [0]
- A little [1]
- Moderately [2]
- A lot [3]
- Not applicable [4]

41.9 disc_findjob

Have you been treated unfairly in finding a job? (this means finding full or part-time paid work)

Expects a single option response (**required**)

- Not at all [0]
- A little [1]
- Moderately [2]
- A lot [3]
- Not applicable [4]

41.10 disc_keepjob

Have you been treated unfairly in keeping a job?

Expects a single option response (**required**)

- Not at all [0]
- A little [1]
- Moderately [2]
- A lot [3]
- Not applicable [4]

41.11 disc_transport

Have you been treated unfairly when using public transport? [Ask about using free travel pass, passengers, drivers, etc]

Expects a single option response (**required**)

- Not at all [0]
- A little [1]
- Moderately [2]
- A lot [3]
- Not applicable [4]

41.12 disc_welfare

Have you been treated unfairly in getting welfare benefits or disability pensions? [Ask about level of benefits, support, applying for benefits eg income support, disability living allowance]

Expects a single option response (**required**)

- Not at all [0]
- A little [1]
- Moderately [2]
- A lot [3]
- Not applicable [4]

41.13 disc_rel

Have you been treated unfairly in your religious practices? [Ask about attending church, other church members, church leaders]

Expects a single option response (**required**)

- Not at all [0]
- A little [1]
- Moderately [2]
- A lot [3]
- Not applicable [4]

41.14 disc_soc

Have you been treated unfairly in your social life? [Ask about socialising, hobbies, attending events, leisure activities]

Expects a single option response (**required**)

- Not at all [0]
- A little [1]
- Moderately [2]
- A lot [3]
- Not applicable [4]

41.15 disc_pol

Have you been treated unfairly by the police? [Ask about any contact with police because of mental health problems or any other reasons]

Expects a single option response (**required**)

- Not at all [0]
- A little [1]
- Moderately [2]
- A lot [3]
- Not applicable [4]

41.16 disc_phys

Have you been treated unfairly when getting help for physical health problems? [Ask about GP, dentist, nurses and emergency treatment including A&E]

Expects a single option response (**required**)

- Not at all [0]
- A little [1]
- Moderately [2]
- A lot [3]
- Not applicable [4]

41.17 disc_mental

Have you been treated unfairly by mental health staff? [Ask about treatment and behaviour of staff, feeling disrespected or humiliated by contact with mental health staff]

Expects a single option response (**required**)

- Not at all [0]
- A little [1]
- Moderately [2]
- A lot [3]
- Not applicable [4]

41.18 disc_privacy

Have you been treated unfairly in your levels of privacy? [Ask about privacy in hospital and in community settings, eg private letters or phone calls, medical records, Criminal Records Bureau check]

Expects a single option response (**required**)

- Not at all [0]
- A little [1]
- Moderately [2]
- A lot [3]
- Not applicable [4]

41.19 disc_safe

Have you been treated unfairly in your personal safety and security? [Ask about verbal abuse, physical abuse, assault]

Expects a single option response (**required**)

- Not at all [0]
- A little [1]
- Moderately [2]
- A lot [3]
- Not applicable [4]

41.20 disc_child

Have you been treated unfairly in starting a family or having children? [Ask about the behaviour of health professionals, friends and family, as well as how they or their partner were treated during pregnancy or childbirth]

Expects a single option response (**required**)

- Not at all [0]
- A little [1]
- Moderately [2]
- A lot [3]
- Not applicable [4]

41.21 disc_parent

Have you been treated unfairly in your role as a parent to your children? [Ask about behaviour of other parents, teachers, family or mental health staff]

Expects a single option response (**required**)

- Not at all [0]
- A little [1]
- Moderately [2]
- A lot [3]
- Not applicable [4]

41.22 disc_avoid

Have you been avoided or shunned by people who know that you have a mental health problem?

Expects a single option response (**required**)

- Not at all [0]
- A little [1]
- Moderately [2]
- A lot [3]
- Not applicable [4]

Section Prerequisites
Skip when ***participant (3.3)*** **Equals'Patient [2]'** OR
Skip when ***cohort (3.2)*** **Equals'epilepsy [5]'** OR
Skip when ***cohort (3.2)*** **Equals'maternal depression [2]'**

Section 42. WHOFIS-Stigma

Prerequisites
Skip when ***country (3.1)*** **Equals 'South Africa - Dr KK [4]'**

42.1 Stigma Instructions 1

Families can have a lot of different experiences when one of its members has psychiatric problems. Can you please tell me whether any of the following things have happened- not at all, sometimes, often or a lot in the past SIX months.

Prerequisites
Skip when ***country (3.1)*** **Not Equal 'South Africa - Dr KK [4]'**

42.2 Stigma Instructions 1_SA

Families can have a lot of different experiences when one of its members has psychiatric problems. Can you please tell me whether any of the following things have happened- not at all, sometimes, often or a lot in the past SIX months. [Show FLASH CARD 7]

42.3 treat

You worried that your neighbours would treat you differently

Expects a single option response (**required**)

- Not at all [0]
- Sometimes [1]
- Often [2]
- A lot [3]

42.4 findout

You spent time worrying whether people would find out about it

Expects a single option response (**required**)

- Not at all [0]
- Sometimes [1]
- Often [2]
- A lot [3]

42.5 hide

You sometimes felt the need to hide this fact

Expects a single option response (**required**)

- Not at all [0]
- Sometimes [1]
- Often [2]
- A lot [3]

42.6 helpothers

You have helped other people to understand what it is like to have a family member with psychiatric problems

Expects a single option response (**required**)

- Not at all [0]
- Sometimes [1]
- Often [2]
- A lot [3]

42.7 secret

When you met people for the first time, you made a special effort to keep this fact a secret

Expects a single option response (**required**)

- Not at all [0]
- Sometimes [1]
- Often [2]
- A lot [3]

42.8 frndfamavoid

You worried that friends and neighbours would avoid you after they found out about it

Expects a single option response (**required**)

- Not at all [0]
- Sometimes [1]
- Often [2]
- A lot [3]

42.9 explain

You have found yourself explaining to others that __(name)__ isnt like their picture of crazy people

Expects a single option response (**required**)

- Not at all [0]
- Sometimes [1]
- Often [2]
- A lot [3]

42.10 blame

You worried that people would blame you for his or her problems

Expects a single option response (**required**)

- Not at all [0]
- Sometimes [1]
- Often [2]
- A lot [3]

42.11 marry

You worried that a person looking to marry would be reluctant to marry into your family

Expects a single option response (**required**)

- Not at all [0]
- Sometimes [1]
- Often [2]
- A lot [3]

42.12 out

You worried about taking him or her out

Expects a single option response (**required**)

- Not at all [0]
- Sometimes [1]
- Often [2]
- A lot [3]

42.13 embarrass

You felt ashamed or embarrassed about it

Expects a single option response (**required**)

- Not at all [0]
- Sometimes [1]
- Often [2]
- A lot [3]

42.14 sought

You sought out people who also have a family member who has psychiatric problems

Expects a single option response (**required**)

- Not at all [0]
- Sometimes [1]
- Often [2]
- A lot [3]

42.15 grief

You felt grief or depression because of it

Expects a single option response (**required**)

- Not at all [0]
- Sometimes [1]
- Often [2]
- A lot [3]

42.16 fault

- A lot [3]

42.15 grief

You felt grief or depression because of it

Expects a single option response (**required**)

- Not at all [0]
- Sometimes [1]
- Often [2]

- A lot [3]

42.16 fault

You felt somehow it might be your fault

Expects a single option response (**required**)

- Not at all [0]
- Sometimes [1]
- Often [2]
- A lot [3]

Section Prerequisites
Skip when ***participant (3.3)*** **Equals'Patient [2]'** OR
Skip when ***cohort (3.2)*** **Equals'epilepsy [5]'** OR
Skip when ***cohort (3.2)*** **Equals'maternal depression [2]'**

Section 43. Caregiver Work Burden

43.1 reducework

In the past one month, has __{376541}__ had to stop or reduce work or other activities because of his/her ill health?

Expects a single option response (**required**)

- No [0]
- Yes [1]

Branches

If response **Equals 'No [0]'** then skip to ***frndfam_reducework (43.6)***

43.2 reduceworkd

How many days in the past one month, did __{376541}__ reduce work/activities? [If answer in HOURS/WEEK, enter 0 to skip ahead]

Expects a numeric response (**required**)

43.3 reduceworkh

How many hours per week was work or activities reduced?

Expects a numeric response (**required**)

43.4 typework

What kind of work did __{376541}__ stop?

Expects a single option response (**required**)

- Housework, that is not paid for (for example housework) [1]
- Manual work (for example farm work or factory work) [2]
- Office worker (for example skills, worker, business, doctor, lawyer) [3]

43.5 moneylost

How much money was lost every day? (before tax and other deductions)

Expects a decimal response (**required**)

43.6 frndfam_reducework

In the past one month, have any friends or family of __{376541}__ had to stop or reduce work or other activities because of his/her ill health?

Expects a single option response (**required**)

- No [0]
- Yes [1]

Branches

If response **Equals 'No [0]'** then skip to ***endofcaregiverworkburden (43.16)***

43.7 frndfam_relation

What is the relationship of this person to __{376541}__?

Expects a single option response (**required**)

- Patient him/herself [1]
- Spouse/partner [2]
- Sibling [3]
- Parent/grandparents/aunt/uncle [4]
- Child, niece/nephew [5]
- Friend [6]
- Other [77]

43.8 frndfam_reduceworkd

How many days in the past one month, did the __{LABEL(376520,__{376520}__)}__ help you reduce work/activities? [If answer is in HOURS/WEEK, enter 0 to skip ahead]

Expects a numeric response (**required**)

43.9 frndfam_reduceworkh

How many hours per week was work or activities reduced?

Expects a numeric response (**required**)

43.10 frndfam_typework

What kind of work did the __{LABEL(376520,__{376520}__)}__ stop?

Expects a single option response (**required**)

- Housework, that is not paid for (for example housework) [1]
- Manual work (for example farm work or factory work) [2]
- Office worker (for example skills, worker, business, doctor, lawyer) [3]

43.11 ofrndfam

In the past one month, have any other friends or family of __{376541}__ had to stop or reduce work or other activities because of his/her ill health?

Expects a single option response (**required**)

- No [0]
- Yes [1]

Branches

If response **Equals 'No [0]'** then skip to ***endofcaregiverworkburden (43.16)***

43.12 ofrndfam_relation

What is the relationship of this person to __{376541}__?

Expects a single option response (**required**)

- Patient him/herself [1]
- Spouse/partner [2]
- Sibling [3]
- Parent/grandparents/aunt/uncle [4]
- Child, niece/nephew [5]
- Friend [6]
- Other [77]

43.13 ofrndfam_reduceworkd

How many days in the past one month, did the __{LABEL(376525,__{376525}__)}__ reduce work/activities? [If answer is in HOURS/WEEK, enter 0 to skip ahead]

Expects a numeric response (**required**)

43.14 ofrndfam_reduceworkh

How many hours per week was work or activities reduced?

Expects a numeric response (**required**)

43.15 ofrndfam_typework

What kind of work did the __{LABEL(376525,__{376525}__)}__ stop?

Expects a single option response (**required**)

- Housework, that is not paid for (for example housework) [1]
- Manual work (for example farm work or factory work) [2]
- Office worker (for example skills, worker, business, doctor, lawyer) [3]

43.16 endofcaregiverworkburden

[End of section]

Section Prerequisites
Skip when ***participant (3.3)*** **Equals'Patient [2]'** OR
Skip when ***cohort (3.2)*** **Equals'epilepsy [5]'** OR
Skip when ***cohort (3.2)*** **Equals'maternal depression [2]'**

Section 44. WHOFIS-Impact

44.1 fisinstruction

Now I would like to ask some specific questions about your own involvement with __{376541}__ and the involvement of other family members over the past SIX months.

44.2 depends

Would you say that you are the family member that __{376541}__ depends on the most?

Expects a single option response (**required**)

- No [0]
- Yes [1]

44.3 involved

Overall, how much have you been involved in helping __{376541}__ when he/she has had psychiatric problems?

Expects a single option response (**required**)

- Not at all [0]
- A little [1]
- Some [2]
- A lot [3]

44.4 fis instruction 2

I would now like to ask you some questions about how __{376541}__'s problems may have affected your life in the past or might in the future.

44.5 social_past

Have __{376541}__ problems made it difficult for you to have the type of social life you would like to have? E.g. caused you to stay at home when you would have liked to be visiting friends [IN THE PAST]

Expects a single option response (**required**)

- Not at all [0]
- A little [1]
- Some [2]
- A lot [3]
- Not applicable [9]

44.6 social_future

Have __{376541}__'s problems made it difficult for you to have the type of social life you would like to have? E.g. caused you to stay at home when you would have liked to be visiting friends [MIGHT IN THE FUTURE]

Expects a single option response (**required**)

- Not at all [0]
- A little [1]
- Some [2]
- A lot [3]
- Not applicable [9]

44.7 strain_past

Have __{376541}__'s problems caused strain between you and other family members? E.g. disagreements over who should care for [the patient] [IN THE PAST]

Expects a single option response (**required**)

- Not at all [0]
- A little [1]
- Some [2]
- A lot [3]
- Not applicable [9]

44.8 strain_future

Have __{376541}__'s problems caused strain between you and other family members? E.g. disagreements over who should care for [the patient's] [MIGHT IN THE FUTURE]

Expects a single option response (**required**)

- Not at all [0]
- A little [1]
- Some [2]
- A lot [3]
- Not applicable [9]

44.9 typework_past

Have __{376541}__'s problems made it difficult for you to do the type of work you would like to do? E.g. turned down job opportunities or did not work because of the need to take care of [the patient's] [IN THE PAST]

Expects a single option response (**required**)

- Not at all [0]
- A little [1]
- Some [2]
- A lot [3]
- Not applicable [9]

44.10 typework_future

Have __{376541}__'s problems made it difficult for you to do the type of work you would like to do? E.g. turned down job opportunities or did not work because of the need to take care of [the patient's] [MIGHT IN THE FUTURE]

Expects a single option response (**required**)

- Not at all [0]
- A little [1]
- Some [2]
- A lot [3]
- Not applicable [9]

44.11 finance_past

Have __{376541}__'s problems caused you financial difficulties? E.g. forced you to devote scarce resources to [the patient's] care [IN THE PAST]

Expects a single option response (**required**)

- Not at all [0]
- A little [1]
- Some [2]
- A lot [3]
- Not applicable [9]

44.12 finance_future

Have __{376541}__'s problems caused you financial difficulties? E.g. forced you to devote scarce resources to [the patient's] care [MIGHT IN THE FUTURE]

Expects a single option response (**required**)

- Not at all [0]
- A little [1]
- Some [2]
- A lot [3]
- Not applicable [9]

Section Prerequisites
Skip when ***participant (3.3)*** **Equals'Patient [2]'** OR
Skip when ***cohort (3.2)*** **Equals'epilepsy [5]'** OR
Skip when ***cohort (3.2)*** **Equals'maternal depression [2]'**

Section 45. Human Rights Abuse - Caregiver

Prerequisites
Skip when ***visit (3.7)*** **Not Equal 'Baseline [1]'**

45.1 hrcgever

Has __{376541}__ ever been chained, restrained or confined?

Expects a single option response (**required**)

- No [0]
- Yes [1]

Branches

If response **Equals 'No [0]'** then skip to ***endofhumanrightssection (45.7)***

Prerequisites
Skip when ***visit (3.7)*** **Not Equal 'Baseline [1]'**

45.2 hrcgeverwhom

By whom? Anyone else?

Expects multiple selected options (**required**)

- Traditional healer [0]
- Religious healer [1]
- Health care worker [2]
- Family member [3]
- Prison staff or police [4]
- Other community member [5]
- Other [77]

Prerequisites
Skip when ***hrcgeverwhom (45.2)*** **Excludes 'Other [77]'**

45.3 hrcgeverwhomo

Please specify who chained, restrained or confined __{376541}__

Expects a single line text response (**required**)

45.4 hrcgmonth

Has __{376541}__ been chained, restrained or confined in the last ONE month?

Expects a single option response (**required**)

- No [0]
- Yes [1]

Branches

If response **Equals 'No [0]'** then skip to ***endofhumanrightssection (45.7)***

45.5 hrcgmonthwhom

By whom? Anyone else?

Expects multiple selected options (**required**)

- [ ] Traditional healer [0]
- [ ] Religious healer [1]
- [ ] Health care worker [2]
- [ ] Family member [3]
- [ ] Prison staff or police [4]
- [ ] Other community member [5]
- [ ] Other [77]

Prerequisites
Skip when *hrcgmonthwhom (45.5)* Excludes 'Other [77]'

45.6 hrcgmonthwhomo

Please specify who chained, restrained or confined __{376541}__

Expects a single line text response (**required**)

45.7 endofhumanrightssection

[End of human rights section]

Section Prerequisites
Skip when *visit (3.7)* Equals'Midline [2]'

Section 46. socioeconomics

Prerequisites
Skip when *country (3.1)* Equals 'South Africa - Dr KK [4]'

46.1 sesinstructions

[This section is a placeholder for all countries. In the event that a participant does not provide informed consent to EMERALD, we need to collect socioeconomic status data in the PRIME interview. This section will contain 10 country-specific questions about household quality, water source, fuel use, toilet and assets. These questions will be a subset of questions used in the EMERALD questionnaire.]

46.2 Household Index Instruction

I want to ask you a few questions about the characteristics of your home.

46.3 tvradio

Does your household have a television or radio?

Expects a single option response (**required**)

- Yes [1]
- No [0]

Prerequisites
Skip when *country (3.1)* Equals 'Uganda - Kamuli [5]'

46.4 hotplate

Does your household have a 2-plate hotplate?

Expects a single option response (**required**)

- No [0]
- Yes [1]

46.5 FRIGE

Does your household have a refrigerator?

Expects a single option response (**required**)

- No [0]
- Yes [1]

Prerequisites
Skip when *country (3.1)* Equals 'Uganda - Kamuli [5]'

46.6 microwave

Does your household have a microwave?

Expects a single option response (**required**)

- No [0]
- Yes [1]

Prerequisites
Skip when *country (3.1)* Equals 'Uganda - Kamuli [5]'

46.7 stove

Does your household have a stove (hotplate with oven)?

Expects a single option response (**required**)

- No [0]
- Yes [1]

46.8 dstv

Does your household have DSTV or GOTV [for Uganda]?

Expects a single option response (**required**)

- No [0]
- Yes [1]

46.9 fan

Does your household have a fan?

Expects a single option response (**required**)

- No [0]
- Yes [1]

Prerequisites
Skip when ***country (3.1)*** **Equals 'Uganda - Kamuli [5]'**

46.10 washing

Does your household have a washing machine?

Expects a single option response (**required**)

- No [0]
- Yes [1]

Prerequisites
Skip when ***country (3.1)*** **Equals 'South Africa - Dr KK [4]'**

46.11 sofa

Does your household have a sofa set?

Expects a single option response (**required**)

- No [0]
- Yes [1]

46.12 heater

Does your household have a heater?

Expects a single option response (**required**)

- No [0]
- Yes [1]

Prerequisites
Skip when ***country (3.1)*** **Equals 'South Africa - Dr KK [4]'**

46.13 bicycle

Does your household have a bicycle?

Expects a single option response (**required**)

- No [0]
- Yes [1]

Prerequisites
Skip when ***country (3.1)*** **Equals 'South Africa - Dr KK [4]'**

46.14 motocycle

Does your household have a motorcycle?

Expects a single option response (**required**)

- No [0]
- Yes [1]

Prerequisites
Skip when ***country (3.1)*** **Equals 'South Africa - Dr KK [4]'**

46.15 goats

Does your household have cows or goats?

Expects a single option response (**required**)

- No [0]
- Yes [1]

46.16 WATER

What is the main source of drinking water for members of your household?

Expects a single option response (**required**)

- Outside shared (Communal) tap [6]
- Tap inside home [7]
- Outside home, own tap [8]
- Outside other source of water [9]

46.17 TOILET

What kind of toilet facility do members of your household usually use?

Expects a single option response (**required**)

- Inside own flush toilet [1]
- Outside shared flush toilet [6]
- Outside own flush toilet [7]
- Outside other toilet (not flush) [8]

46.18 rooms

How many rooms does your household have?

Expects a numeric response (**required**)

46.19 home

What type of home do you live in? Is it a...

Expects a single option response (**required**)

- Shack [1]
- Brick/cement block home [2]
- Flat [3]
- Outbuilding [4]
- Rented room [5]

Section 47. end

Prerequisites
Skip when ***country (3.1)*** **Not Equal 'South Africa - Dr KK [4]'** OR
Skip when ***SUITHINK (10.2)*** **Not Equal 'Yes [1]'**

47.1 suicideref

[TASNEEM - PUT SCRIPT FOR REFERRAL HERE. Suicidal ideation response = Label(q374222 , q374222) Suicidal planning response = Label(q274223 , q374223) Suicidal action response = Label(q374224 , q374224)]

Prerequisites
Skip when ***cohort (3.2)*** **Not Equal 'maternal depression [2]'** OR
Skip when ***TotalPHQ (1.18)*** **Less Than '10'** OR
Skip when ***visit (3.7)*** **Equals 'Baseline [1]'**

47.2 mdref

[This participant has a PHQ-9 score of 10 or above - this means she may be suffering from depression. Please consult with the psychiatric clinical officer for further assessment]

47.3 comments

[Enter any comments here about participant or interview]

Expects a single line text response (optional)

47.4 endofquestionnaire

[End of questionnaire. Thank you.]
